# A hybrid metaheuristic framework for robust photovoltaic model parameter estimation based on crested porcupine and differential evolution algorithms

**DOI:** 10.1038/s41598-026-67591-x

**Published:** 2026-09-02

**Authors:** Ali Ahmed Hussein Mohamed, Ibrahim Mohamed Diaaeldin, Mahmoud A. Attia, Walid Helmy

**Affiliations:** 1 Electrical Power and Machines Department, Faculty of Engineering, Ain Shams University, Cairo, 11517 Egypt; 2 Engineering Physics and Mathematics Department, Ain Shams University, Cairo, 11517 Egypt

**Keywords:** Solar energy systems, Photovoltaic parameter estimation, Triple Diode Model (TDM), Hybrid metaheuristic optimization, Crested Porcupine Optimizer (CPO), Differential Evolution (DE), Newton–Raphson method, Energy science and technology, Engineering, Mathematics and computing

## Abstract

Accurate parameter estimation is essential for the efficient design and performance evaluation of solar photovoltaic (PV) systems. However, the strong nonlinearity of PV equivalent circuit models often causes conventional optimization algorithms to become trapped in local optima. To overcome this challenge, this paper proposes a novel hybrid optimization framework, termed Crested Porcupine Differential Evolution (CPO-DE), which combines the global exploration capability of the Crested Porcupine Optimizer with the robust mutation mechanism of Differential Evolution. The proposed CPO-DE algorithm is first validated mathematically using 23 benchmark functions alongside three classical engineering design problems. It is subsequently applied to the Single Diode Model (SDM), Double Diode Model (DDM), and Triple Diode Model (TDM) of PV systems. In addition, a novel two-stage evaluation strategy is introduced, where computationally efficient algebraic equations are employed during the iterative optimization process to reduce computational burden, followed by an explicit Newton–Raphson numerical solver to ensure high physical accuracy of the estimated parameters. The effectiveness of the proposed approach is evaluated using experimental data from the RTC France solar cell over 300 independent runs. The results demonstrate that CPO-DE achieves a 100% convergence success rate with near-zero standard deviations, indicating excellent robustness and stability. Moreover, the proposed method outperforms eleven state-of-the-art optimization algorithms and consistently attains the top rank according to Friedman statistical tests. These results confirm that CPO-DE is a highly reliable, stable, and efficient engineering tool for advanced photovoltaic model parameter estimation.

**Supplementary Information:**

The online version contains supplementary material available at https://doi.org/10.1038/s41598-026-67591-x.

## Introduction

Massive industrial and technological progress has led to a sharp increase in global energy demand. This surge is especially driven by the rise and continuous expansion of data centers needed to run AI servers^1^. At the same time, the world is actively striving to adopt sustainable technologies and cut carbon emissions. Because of these factors, expanding the use of renewable energy has become an urgent global imperative. Among these sources, solar energy stands out as a highly deployed solution. Compared to other renewable energy sources (RESs), solar photovoltaic (PV) technology has secured the highest level of financial investment lately. According to the IEA, this is driven by its massive contribution to the global green energy mix, generating over 75% of the world's renewable power^2^, while naturally producing zero carbon emissions^3^.

Despite solar energy's clear environmental benefits, significant engineering challenges remain before it can reach its maximum efficiency. Accurate mathematical models are essential to simulate and optimize the behavior of solar cells. However, precise parameter estimation is a highly complex process, primarily because manufacturers only provide standard nominal data. Without identifying these internal variables under real operating conditions, maximizing the power output of PV systems remains unattainable.

The mathematical models that describe the electrical behavior of a solar cell involve highly non-linear equations. These models are deeply affected by fluctuating weather conditions, solar radiation, and ambient temperature, highlighting the critical impact of thermal effects and partial shading on the overall characteristics of solar panels^4,5^. Consequently, recent research has placed significant emphasis on increasing the accuracy of solar cell performance models^6–8^.

The single diode model (SDM) is the most widely utilized baseline model^9^, which describes the current–voltage characteristics for a PV cell by estimating five unknown parameters. While useful, the SDM has a physical limitation: it assumes that recombination losses in the depletion region are negligible. This simplification reduces its accuracy, particularly under low solar irradiance conditions. To overcome this fundamental drawback, the double-diode model (DDM) was extensively adopted^10^. By adding a second parallel diode to account for recombination losses, the DDM provides a highly accurate reflection of true cell behavior, though it increases the total unknown variables from five to seven. Despite these advancements, the DDM still struggles to accurately capture several complex physical phenomena, such as large leakage currents and recombination losses in the grain boundaries and defect regions. To address these limitations and precisely replicate this highly non-linear behavior, the triple-diode model (TDM) is widely adopted^11^. Incorporating a third diode offers the highest degree of precision and reliability, particularly during significant temperature swings and fluctuating irradiance levels^12^. Because it necessitates the estimation of nine unknown parameters, this high level of precision demands a high computational cost. Traditional estimation techniques find it very challenging to solve this multidimensional problem, hence the TDM is purposefully included in this work as a highly challenging benchmark.

Accurately determining these internal parameters is crucial to assess PV cell performance and guarantee the dependable design of large-scale solar systems. Accordingly, research on PV parameter estimation methods has accelerated tremendously over the past decade. The methodologies used to solve this problem generally fall into three primary categories: analytical approaches, deterministic numerical methods, and evolutionary meta-heuristic algorithms^13,14^.

Recent literature highlights a significant shift towards these advanced computational models. While traditional analytical and numerical methods continue to be rigorously evaluated for basic equivalent circuits^15^, evolutionary metaheuristics have become the standard for overcoming the complex nonlinearities of PV systems. For instance, numerous studies have successfully deployed novel, standalone nature-inspired algorithms to accurately extract parameters across varying environmental conditions^16–19^. More recently, innovative paradigms have been introduced to push the boundaries of estimation accuracy, such as the sport-inspired Kabaddi Game Optimizer (KGO)^20^ and the nature-inspired Marine Walrus Optimization (MWO) algorithm^21^, both demonstrating highly competitive performance in diode model extractions. Furthermore, to enhance search stability and effectively balance exploration with exploitation, recent research focus has increasingly shifted toward developing robust hybrid metaheuristic frameworks^22–24^, a trend that strongly aligns with the core motivation of this study.

Analytical methods typically rely on mathematical equations combined with manufacturer datasheets or in-field measurements. Various analytical techniques have been proposed, including nonlinear least-squares fitting^25^, statistical curve fitting^26^, and point-based analytical estimation approaches^27^. Comprehensive reviews, such as those evaluating techniques on the Kyocera KC200GT module^28^, demonstrate their practical utility. While the analytical approach is valued for its simplicity and rapid computation, its reliance on necessary mathematical assumptions inevitably limits the overall precision of the estimated parameters. To overcome these limitations, numerical iterative methodologies were proposed.

Deterministic numerical methods have been widely utilized in the literature, including the Newton–Raphson method^29^, Lambert W functions^30,31^, the Berndt–Hall–Hall–Hausman algorithm^32^, the Levenberg–Marquardt Algorithm )LMA)^33^ , and comparative numerical approaches evaluating the Villalva, Wang, and Vika methods^34^. Although comparative studies indicate that iterative curve fitting via the Newton–Raphson method yields higher accuracy than Lambert W approximations, these numerical techniques encounter specific mathematical challenges. Due to the highly non-linear nature of solar cell equations, gradient-based solvers are inherently sensitive to initial parameter guesses and may occasionally converge to local optima. Furthermore, the reliance on continuous derivative calculations increases the computational burden, making it difficult to seamlessly scale these methods to higher-dimensional models like the TDM.

To bypass the restrictions of deterministic techniques, researchers have actively turned to meta-heuristic optimization algorithms^35,36^. These evolutionary techniques are derivative-free, simple to implement, and highly effective. They exhibit a strong ability to fully explore the search space and avoid local optima, yielding high precision when estimating the parameters of complex PV models. Recently, researchers have relied heavily on these algorithms—spanning evolutionary, physics-inspired, swarm intelligence, and human-behavior-based families^7^, and their elegant hybrid versions. However, according to the "No Free Lunch" (NFL) theorem^37^, no single optimization method can consistently and equally address all engineering problems. An algorithm that successfully solves a basic SDM may fail or stagnate when applied to a more complicated, multidimensional problem like the TDM.

Consequently, developing robust optimization algorithms capable of navigating complex PV landscapes to ensure the highest level of accuracy remains a critical research gap. While the Crested Porcupine Optimizer (CPO)^38^ offers excellent global exploration, it is susceptible to stagnation in local optima when navigating high-dimensional PV models. To overcome this limitation, this paper proposes a novel, highly robust hybrid optimization method, CPO-DE. This approach strategically integrates the global search power of CPO with the stable mutation mechanisms of Differential Evolution (DE)^39^, effectively preventing premature convergence. The primary objective of this study is to develop a robust, highly accurate, and stable hybrid optimization framework (CPO-DE) to reliably estimate the parameters of complex PV models. Unlike traditional studies that merely focus on achieving marginally lower mathematical errors, this work is motivated by the need to resolve structural algorithmic vulnerabilities when dealing with highly complex models. To achieve this, the specific sub-objectives and main contributions of this work are highlighted as follows:

- A Novel Hybrid Framework: A highly stable optimization algorithm (CPO-DE) is proposed, uniquely designed to maintain an optimal balance between exploration and exploitation. By substituting the destructive physical attack phase of standard CPO with a DE-based mutation strategy, the proposed framework successfully solves the stagnation vulnerability of the standard CPO.
- Mathematical Validation: Prior to real-world application, the mathematical robustness of CPO-DE is rigorously validated across 23 standard benchmark test functions, proving its superior search capabilities in diverse multi-dimensional landscapes.
- Comprehensive PV Application: The proposed framework is successfully utilized to estimate the intricate parameters of solar cells across three progressively complex equivalent circuits: SDM, DDM, and the highly non-linear 9-dimensional TDM.
- Robust Statistical Outperformance: The performance of CPO-DE is comprehensively evaluated against eleven foundational and state-of-the-art algorithms. Rigorous statistical tests (Wilcoxon and Friedman) confirm its superior performance, exhibiting near-zero variance and high consistency across all runs, proving that the proposed approach guarantees absolute statistical reliability rather than just peak accuracy.
- Exact Physical Precision: By integrating a robust evaluation strategy, the parameter estimation by CPO-DE demonstrates high physical precision. Rather than merely fitting a mathematical curve, the results closely match the simulated I-V and P-V characteristic curves with the actual experimental data of the RTC France solar cell.

The remaining sections of this paper are organized as follows: "PV modelling approaches" section provides the mathematical formulation of the three equivalent PV models and the design of the objective function. "Methodology" section details the structure, mechanisms, and the novel two-stage evaluation strategy of the proposed hybrid CPO-DE algorithm. "Results and discussion" section presents a comprehensive discussion of the simulation results, encompassing the mathematical benchmark validations, statistical analysis, and the core application of PV parameter estimation for the SDM, DDM, and TDM. Finally, "Conclusions" section concludes the work and suggests potential directions for future research.

## PV modelling approaches

This section details the mathematical formulations of the SDM, DDM, and TDM, and defines the parameter estimation problem required to accurately establish these PV models. It is important to note that these models are employed in this study as increasingly challenging optimization testbeds rather than novel physical proposals. The transition from the basic 5-parameter SDM to the highly non-linear 9-parameter TDM is technically significant to capture complex physical phenomena (e.g., grain boundary recombinations and large leakage currents) while rigorously testing the algorithm's scalability and robustness against local optima stagnation. Although higher-order models (e.g., four-diode models) exist theoretically, the TDM is purposefully selected as the ultimate benchmark in this work, as it offers the optimal balance between ultra-high physical precision and practical computational viability.

### Single Diode Model (SDM)

**Fig. 1:**
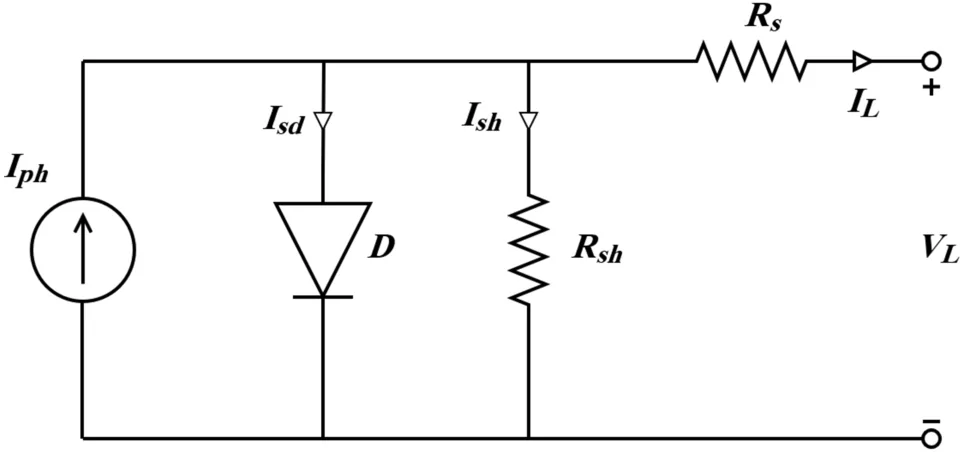
Equivalent circuit of single-diode model.

The Single Diode Model (SDM) is the most fundamental equivalent circuit used to describe the electrical behavior of a PV cell. The equivalent circuit of the single-diode model (SDM) is shown in Fig. 1^40^. The following Kirchhoff’s Current Law (KCL) Eq. (1)^41^ can be used to represent the transcendental equation produced in the SDM:

1 $${I}_{L}={I}_{ph}-{I}_{D}-{I}_{sh},$$

By substituting the mathematical expressions for the diode current $\left({I}_{D}\right)$ and the shunt leakage current $\left({I}_{sh}\right)$, the complete transcendental formulation is given by^42^:

2 $${I}_{L}={I}_{ph}-{I}_{sd}\left[exp\left(\frac{{V}_{L}+{I}_{L}\cdot {R}_{s}}{n\cdot {V}_{t}}\right)-1\right]-\frac{{V}_{L}+{I}_{L}\cdot {R}_{s}}{{R}_{sh}},$$

The thermal voltage ${V}_{t}$ is defined as follows^42^:

3 $${V}_{t}=\frac{k\cdot T}{q},$$

where:

- ${I}_{ph}, {I}_{sd}:$ represent the photo-generated current and the diode reverse saturation current, respectively,
- *n:* is the diode ideality factor,
- ${R}_{s}, {R}_{sh}:$ are the series and shunt resistances of the equivalent circuit, respectively,
- ${V}_{L},{I}_{L}:$ denote the output terminal voltage and the load current,
- $k=1.38\times {10}^{-23}J/K$ is the Boltzmann constant,
- *T* is the working temperature in Kelvin,
- $q=1.602\times {10}^{-19} C$ is the absolute value of electron charge.

To simulate the SDM, five unknown internal parameters must be accurately estimated by the optimization algorithm. These parameters forming the decision vector $\left(x\right)$^41^ are as follows:

4 $$x=\left[{I}_{ph},{I}_{sd},n,{R}_{s},{R}_{sh}\right].$$

### Double Diode Model (DDM)

**Fig. 2:**
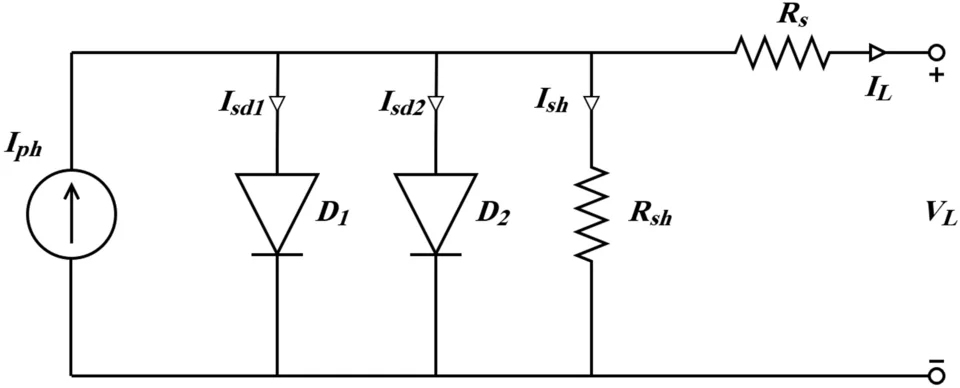
Equivalent circuit of double-diode model.

The Double-Diode Model (DDM) is introduced to overcome the physical constraints of the SDM, especially in low solar irradiation situations as shown in Fig. 2^43^. A second diode is introduced to represent the recombination current losses occurring within the depletion region of the solar cell. By applying Kirchhoff’s Current Law (KCL) to the DDM equivalent circuit, the output load current $\left({I}_{L}\right)$ is expressed in Eq. (5)^41^, while the diodes and the shunt resistor currents are defined by Eqs. (6) – (8)^42^:

5 $${I}_{L}={I}_{ph}-{I}_{D1}-{I}_{D2}-{I}_{sh},$$

6 $${I}_{D1}={I}_{sd1}\left[exp\left(\frac{{V}_{L}+{I}_{L}\cdot {R}_{s}}{{n}_{1}\cdot {V}_{t}}\right)-1\right],$$

7 $${I}_{D2}={I}_{sd2}\left[exp\left(\frac{{V}_{L}+{I}_{L}\cdot {R}_{s}}{{n}_{2}\cdot {V}_{t}}\right)-1\right],$$

8 $${I}_{sh}=\frac{{V}_{L}+{I}_{L}\cdot {R}_{s}}{{R}_{sh}},$$

where:

- ${I}_{D1}, {I}_{D2}$ are the first and second diode currents, respectively,
- ${I}_{sd1}, {I}_{sd2}$ are the diffusion and recombination reverse saturation current,
- ${n}_{1}, {n}_{2}$ are the diffusion and recombination diode ideality factors,
- ${I}_{sh}$ is the leakage current passing through the shunt resistor.

By substituting Eqs. (6, 7, 8) into Eq. (5), the cell output load current can be rewritten as in Eq. (9)^42^.

9 $${I}_{L}={I}_{ph}-{I}_{sd1}\left[exp\left(\frac{{V}_{L}+{I}_{L}\cdot {R}_{s}}{{n}_{1}\cdot {V}_{t}}\right)-1\right]-{I}_{sd2}\left[exp\left(\frac{{V}_{L}+{I}_{L}\cdot {R}_{s}}{{n}_{2}\cdot {V}_{t}}\right)-1\right]-\frac{{V}_{L}+{I}_{L}\cdot {R}_{s}}{{R}_{sh}},$$

As a result, simulating the DDM includes a higher degree of complexity as it has seven unknown internal parameters. The corresponding decision vector $\left(x\right)$^41^ to be optimized is defined as:

10 $$x=\left[{I}_{ph},{I}_{sd1},{I}_{sd2},{n}_{1},{n}_{2},{R}_{s},{R}_{sh}\right].$$

### Triple Diode Model (TDM)

**Fig. 3:**
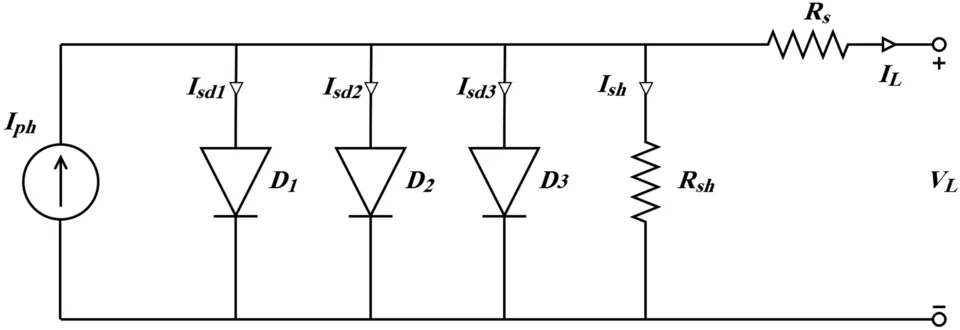
The Triple-Diode Model's equivalent circuit.

The Double Diode Model (DDM) often struggles to accurately capture the highly non-linear behavior of solar cells under severe conditions. Specifically, it has difficulty modeling significant leakage currents and recombination losses that occur in defective areas and grain boundaries. To address these limitations and accurately model these physical effects, the Triple Diode Model (TDM) was introduced. As illustrated in Fig. 3^44^, the TDM incorporates a third diode connected in parallel with the other two diodes and the current source^11^.

By applying Kirchhoff’s Current Law (KCL) to the TDM equivalent circuit, the output load current $\left({I}_{L}\right)$ is expressed in Eq. (11)^41^:

11 $${I}_{L}={I}_{ph}-{I}_{D1}-{I}_{D2}-{I}_{D3}-{I}_{sh},$$

The following is a mathematical definition of the currents passing through the shunt resistor and the three parallel diodes:

12 $${I}_{D1}={I}_{sd1}\left[exp\left(\frac{{V}_{L}+{I}_{L}\cdot {R}_{s}}{{n}_{1}\cdot {V}_{t}}\right)-1\right],$$

13 $${I}_{D2}={I}_{sd2}\left[exp\left(\frac{{V}_{L}+{I}_{L}\cdot {R}_{s}}{{n}_{2}\cdot {V}_{t}}\right)-1\right], $$

14 $${I}_{D3}={I}_{sd3}\left[exp\left(\frac{{V}_{L}+{I}_{L}\cdot {R}_{s}}{{n}_{3}\cdot {V}_{t}}\right)-1\right], $$

15 $${I}_{sh}=\frac{{V}_{L}+{I}_{L}\cdot {R}_{s}}{{R}_{sh}},$$

By substituting Eqs. (12) - (15)^42^ into the main KCL Eq. (11), the complete implicit formulation for the TDM is given by:

16 $${I}_{L}={I}_{ph}-{I}_{sd1}\left[exp\left(\frac{{V}_{L}+{I}_{L}\cdot {R}_{s}}{{n}_{1}\cdot {V}_{t}}\right)-1\right]-{I}_{sd2}\left[exp\left(\frac{{V}_{L}+{I}_{L}\cdot {R}_{s}}{{n}_{2}\cdot {V}_{t}}\right)-1\right]-{I}_{sd3}\left[exp\left(\frac{{V}_{L}+{I}_{L}\cdot {R}_{s}}{{n}_{3}\cdot {V}_{t}}\right)-1\right]-\frac{{V}_{L}+{I}_{L}\cdot {R}_{s}}{{R}_{sh}},$$

where:

- ${I}_{D1}$ denotes the diffusion and recombination current occurring within the bulk and emitter zones of the P–N junction.
- ${I}_{D2}$ represents the recombination current in the depletion region.
- ${I}_{D3}$ represents the effect of grain boundaries and leakage current.
- ${I}_{sd3}$ represents the reverse saturation current of the third diode, accounting for the leakage and recombination in defective regions.
- ${n}_{3}$ represents the ideality factor of the third diode.

Simulating the TDM is considered a significant optimization challenge due to its complex and multidimensional nature. Nine unknown internal parameters must be accurately and simultaneously estimated. The following defines the related decision vector $\left(x\right)$^41^ that needs to be optimized:

17 $$x=\left[{I}_{ph},{I}_{sd1},{I}_{sd2},{I}_{sd3},{n}_{1},{n}_{2},{n}_{3},{R}_{s},{R}_{sh}\right].$$

## Methodology

This section details the proposed technique for accurate photovoltaic (PV) model parameter estimation. This approach addresses the multimodality and non-linearity issues that arise while modeling solar cells. A novel hybrid metaheuristic algorithm is integrated with a numerical solver (Newton–Raphson) to ensure maximum accuracy.

### Problem formulation

To evaluate the quality of the estimated parameters, the Root Mean Square Error (RMSE) is used as the objective function $f\left(x\right)$ to be minimized by the proposed optimization algorithm. The RMSE is formulated as the root mean square of the difference between the measured and calculated module current data^35^. It is specifically chosen over other absolute or time-domain integral-based metrics (such as ISE, ITAE, or IAE) because squaring the residuals heavily penalizes larger discrepancies at individual data points. This mathematical property is crucial in static PV curve fitting, as it forces the algorithm to tightly fit the most critical "knee" region of the I-V curve (the Maximum Power Point). Furthermore, RMSE is the universally accepted standard metric in PV parameter estimation literature, ensuring a fair comparative evaluation. It is mathematically formulated as follows:

18 $$\mathrm{min}RMSE\left(x\right)=\sqrt{\frac{1}{N}\sum_{i=1}^{N}{\left({I}_{meas,i}-{I}_{calc,i}\left({V}_{meas,i},x\right)\right)}^{2}},$$

where:

- *N:* is the total number of experimental data points,
- ${I}_{meas,i}:$ is the *i*-th measured experimental load current,
- ${I}_{calc,i}\left({V}_{meas,i},x\right):$ is the *i*-th simulated load current calculated using the mathematical models [Eqs. (2), (9), or (16)] at the corresponding measured voltage ${V}_{meas,i}$.

Constraints are applied to the optimization problem to guarantee that the calculated parameters fall within the physical range. Every parameter ${x}_{j}$ in the decision vector must satisfy the following condition:

19 $$l{b}_{j}\le {x}_{j}\le u{b}_{j}, j=\mathrm{1,2},\dots ,D,$$

where:

- $l{b}_{j}$ and $u{b}_{j}$ represent the lower and upper bounds of the search space for the *j*-th parameter, respectively,
- *D* is the total number of unknown parameters (dimensions) corresponding to the chosen PV model (*D=5* for SDM, *D=7* for DDM, and *D=9* for TDM).

The upper and lower bounds for the PV parameters were not chosen arbitrarily; rather, they were strictly adopted from widely accepted standard ranges established in foundational PV parameter estimation literature^42,43^ to ensure consistent, physically logical, and fair benchmarking. Table 1 outlines these established boundaries for each parameter optimized in this study.

**Table 1:** The parameters range of the PV SDM, DDM, and TDM.

Parameter	Search range [*lb,ub*]
$${I}_{ph} \left(A\right)$$	[0,1]
${I}_{sd1},{I}_{sd2},{I}_{sd3} \left(A\right)$	[${10}^{-12}$,${10}^{-6}$]
$${R}_{s} \left(\Omega \right)$$	[0,0.5]
$${R}_{sh} \left(\Omega \right)$$	[0,100]
$${n}_{1},{n}_{2},{n}_{3}$$	[1,2]

### Multi-stage optimization

To balance computational efficiency with high physical precision, this study introduces a multi-stage evaluation strategy. Applying the Newton–Raphson (NR) explicit solver iteratively to every search agent during the optimization loop would require millions of complex derivative calculations, creating a significant computational burden. To avoid this, the first stage (during the evolutionary loop) utilizes a fast implicit algebraic residual evaluation to swiftly guide the search process and explore the multi-dimensional space efficiently. In the second stage, the explicit NR solver is applied exclusively to the final global best solution $\left({X}_{best}\right)$. Specifically, the NR method is not used inside the iterative fitness evaluation loop for every candidate solution; rather, it acts solely as a final computational step to solve the implicit PV current equation for the extracted optimal parameters. This strategic separation guarantees that the reported RMSE reflects the exact physical deviation of the solar cell rather than a mere mathematical approximation, while significantly reducing the overall execution time.

#### Mathematical formulation of the Newton–Raphson solver

To apply the NR method, the implicit current equations of the PV models [Eqs. (2), (9), or (16)] must be rewritten as a homogeneous objective function $g\left({I}_{calc}\right)= 0.$ The NR algorithm utilizes the first-order derivative of this function with respect to the current, denoted as ${g}{\prime}\left({I}_{calc}\right)$, to iteratively find the exact root. The mathematical update rule for calculating the simulated current at the $\left(k+1\right)$-th iteration is defined as follows:

20 $${I}_{calc}^{(k+1)}={I}_{calc}^{(k)}-\frac{g({I}_{calc}^{\left(k\right)})}{g{\prime}({I}_{calc}^{\left(k\right)})},$$

where:

- ${I}_{calc}^{\left(k\right)}$ is the calculated current at the current iteration *k*,
- ${I}_{calc}^{(k+1)}$ is the updated current for the next iteration,
- $g\left({I}_{calc}\right)$ is the implicit error function of the specific PV model,
- ${g}{\prime}\left({I}_{calc}\right)$ is the exact analytical derivative of the function *g* with respect to ${I}_{calc}.$

#### Iterative update and convergence criteria

The NR solver requires a precise starting point (initial guess) to prevent divergence. In this study, the generated photocurrent is utilized as the robust initial guess (${I}_{calc}^{\left(0\right)}={I}_{ph}$)^45^. The iterative update process continues until the solver reaches a strictly predefined tolerance limit $\left(\epsilon \right)$, ensuring strict numerical precision. The convergence criterion is mathematically formulated as:

21 $$\left|{I}_{calc}^{(k+1)}-{I}_{calc}^{(k)}\right|\le \epsilon ,$$

where *ε * represents the accuracy threshold, which is strictly set to ${10}^{-10}$ in this study to guarantee a highly accurate explicit solution for the subsequent RMSE evaluation.

### Hybrid crested porcupine differential evolution

This section introduces a new hybrid algorithm called CPO-DE to accurately estimate parameters for complex PV models. It combines the strong exploration of the Crested Porcupine Optimizer (CPO) with the stable mutation of Differential Evolution (DE). Below, we explain the basic math for both algorithms, followed by how we merged them to prevent the algorithm from getting stuck in local optima.

#### Mathematical model of standard crested porcupine optimizer

The Crested Porcupine Optimizer (CPO)^38^ mimics how a porcupine defends itself using four strategies: sight, sound, odor, and physical attack. Sight and sound are used for searching widely (exploration), while odor and physical attacks focus on refining the best solutions (exploitation). The process starts by randomly placing solutions within the search space boundaries:

22 $${X}_{i,j}={lb}_{j}+r\times \left({ub}_{j}-{lb}_{j}\right),$$

where ${X}_{i}$ is the position of the *i*-th individual, ${lb}_{j}$ and ${ub}_{j}$ are the lower and upper bounds of the *j*-th parameter, and *r* is a random number in $\left[\mathrm{0,1}\right]$.

As the algorithm runs, it switches between these strategies. It relies heavily on the "Alpha Porcupine" $\left({X}_{CP}\right)$, which is the most effective solution discovered thus far. Sight and Sound (Exploration Phase): Used when the predator is far. The algorithm searches widely by making large random jumps. Odor and Physical Attack (Exploitation Phase): Used when the predator is nearby. The algorithm quickly moves toward the Alpha Porcupine $\left({X}_{CP}\right)$ using small steps.

Visual Display Strategy:

23 $${X}_{i}^{t+1}={X}_{i}^{t}+\alpha \cdot \left|2\cdot rand\cdot {X}_{best}^{t}-{X}_{i}^{t}\right|,$$

Auditory Warning Strategy:

24 $${X}_{i}^{t+1}={X}_{best}^{t}+\alpha \cdot \left|2\cdot rand\cdot {X}_{best}^{t}-{X}_{i}^{t}\right|\cdot \left(2\cdot rand-1\right),$$

Odor Warning Strategy:

25 $${X}_{i}^{t+1}=\left(1-U\right)\cdot {X}_{i}^{t}+U\cdot \left({X}_{best}^{t}+rand\cdot \left({X}_{r1}^{t}-{X}_{r2}^{t}\right)\right),$$

Physical Attack Strategy:

26 $${X}_{i}^{t+1}={X}_{best}^{t}+\left(2\cdot rand\cdot {S}_{i}-{S}_{i}\right)\cdot \alpha ,$$

where *U* denotes the odor diffusion parameter governing the transition dynamics during the localized search, while ${r}_{1}$ and ${r}_{2}$ represent mutually exclusive random indices chosen from the current population. Furthermore, ${S}_{i}$ characterizes the physical combat strength of the porcupine, and *α * acts as a dynamic defense factor that gradually decreases to balance the exploration and exploitation phases.

Despite the overall efficiency of the CPO, the physical attack phase in Eq. (26) often traps the algorithm in local optima when solving highly complex problems like TDM. To overcome this, a more robust and stable mutation strategy must be integrated.

#### Differential evolution (DE) mechanism

To counteract the premature convergence of the CPO, the Differential Evolution (DE) algorithm^39^ is introduced. DE is robust and effective at maintaining population diversity. It operates through three steps:

Mutation: Instead of picking random starting points, this study uses the stable DE/best/1 strategy. It creates a mutant vector ${V}_{i}^{t}$ based on the best current solution $\left({X}_{best}^{t}\right)$:

27 $${V}_{i}^{t}={X}_{best}^{t}+F\cdot \left({X}_{r1}^{t}-{X}_{r2}^{t}\right),$$

where ${r}_{1}$ and ${r}_{2}$ are mutually exclusive random integer indices distinct from the target index *i* $\left({r}_{1} \ne {r}_{2} \ne i\right)$. The scaling factor *F* controls the amplification of the differential variation.

Crossover: To enhance parameter diversity, a binomial crossover mechanism recombines the mutant vector ${V}_{i}^{t}$ with the target vector ${X}_{i}^{t}$ to generate a trial vector ${U}_{i}^{t}$:

28 $${U}_{i,j}^{t}=\left\{\begin{array}{c}{V}_{i,j}^{t}, if rand\le CR or j={j}_{rand}\\ {X}_{i,j}^{t}, otherwise\end{array},\right.$$

where $CR\in \left[\mathrm{0,1}\right]$ is the crossover rate, *rand* is a uniformly distributed random number, and ${j}_{rand}$ is a randomly chosen parameter index ensuring that at least one dimension is inherited from the mutant vector to prevent stagnation.

Selection (Greedy Strategy): Finally, a greedy selection mechanism compares the fitness of the trial vector ${U}_{i}^{t}$ against the target vector ${X}_{i}^{t}$. The trial vector survives to the next generation only if it yields a superior (lower) objective value (RMSE):

29 $${X}_{i}^{t+1}=\left\{\begin{array}{c}{U}_{i}^{t}, if f\left({U}_{i}^{t}\right)<f\left({X}_{i}^{t}\right)\\ {X}_{i}^{t}, otherwise\end{array}\right.,$$

#### The proposed hybridization strategy

The core concept of the proposed CPO-DE algorithm is to replace the unstable physical attack phase of the standard CPO with the highly robust DE mutation strategy. A stochastic probability mechanism is employed to dictate the update rule for each solution at every iteration. For each search agent ${X}_{i}^{t}$, a uniformly distributed random number $r\in \left[\mathrm{0,1}\right]$ is generated to select one of the following evolutionary paths:

Strategy 1: Wide Search (CPO Visual Sight): If *r < 0.33*, the algorithm explores a broad region of search space using a dynamic defense factor $\left(\upalpha \right)$. The trial vector ${V}_{i}^{t}$ is generated as follows:

30 $${V}_{i}^{t}={X}_{i}^{t}+\alpha \cdot randn\cdot \left({X}_{idx}^{t}-{X}_{i}^{t}\right),$$

where ${X}_{idx}^{t}$ is a random individual from the population, and *randn* is a normal random number. Unlike the standard CPO which relies on the global best in this phase, utilizing a random individual ${X}_{idx}^{t}$ intentionally broadens the global exploration capability and prevents premature convergence.

Strategy 2: Directed Search (CPO Auditory Sound): If *0.33 ≤ r < 0.66*, the algorithm shifts towards exploitation by moving the search agent closer to the current global best solution ${X}_{best}^{t}$:

31 $${V}_{i}^{t}={X}_{i}^{t}+rand\cdot \left({X}_{best}^{t}-{X}_{i}^{t}\right),$$

where *rand* is a uniformly distributed random number in the range $\left[\mathrm{0,1}\right]$.

Strategy 3: Stable Exploitation (DE Mutation): If *r ≥ 0.66*, the algorithm bypasses the standard CPO physical attack to avoid divergence. Instead, it deploys the highly stable DE/best/1 mutation strategy to carefully exploit the promising regions:

32 $${V}_{i}^{t}={X}_{best}^{t}+F\cdot \left({X}_{r1}^{t}-{X}_{r2}^{t}\right),$$

where *F* is the scaling factor, and ${X}_{r1}^{t}$ and ${X}_{r2}^{t}$ are two distinct randomly chosen individuals from the population $\left({r}_{1} \ne {r}_{2} \ne i\right)$.

The Final Mixing Step (Crossover & Selection): Regardless of the strategy used to generate the mutant vector ${V}_{i}^{t}$, the DE binomial crossover Eq. (28) is invariably applied to mix it with the target vector ${X}_{i}^{t}$, generating the trial vector ${U}_{i}^{t}$. This critical filter prevents the algorithm from discarding previously discovered high-quality parameter values. Finally, the greedy selection rule Eq. (29) evaluates the implicit fitness to ensure that only the superior solutions survive into the subsequent generation.

**Fig. 4:**
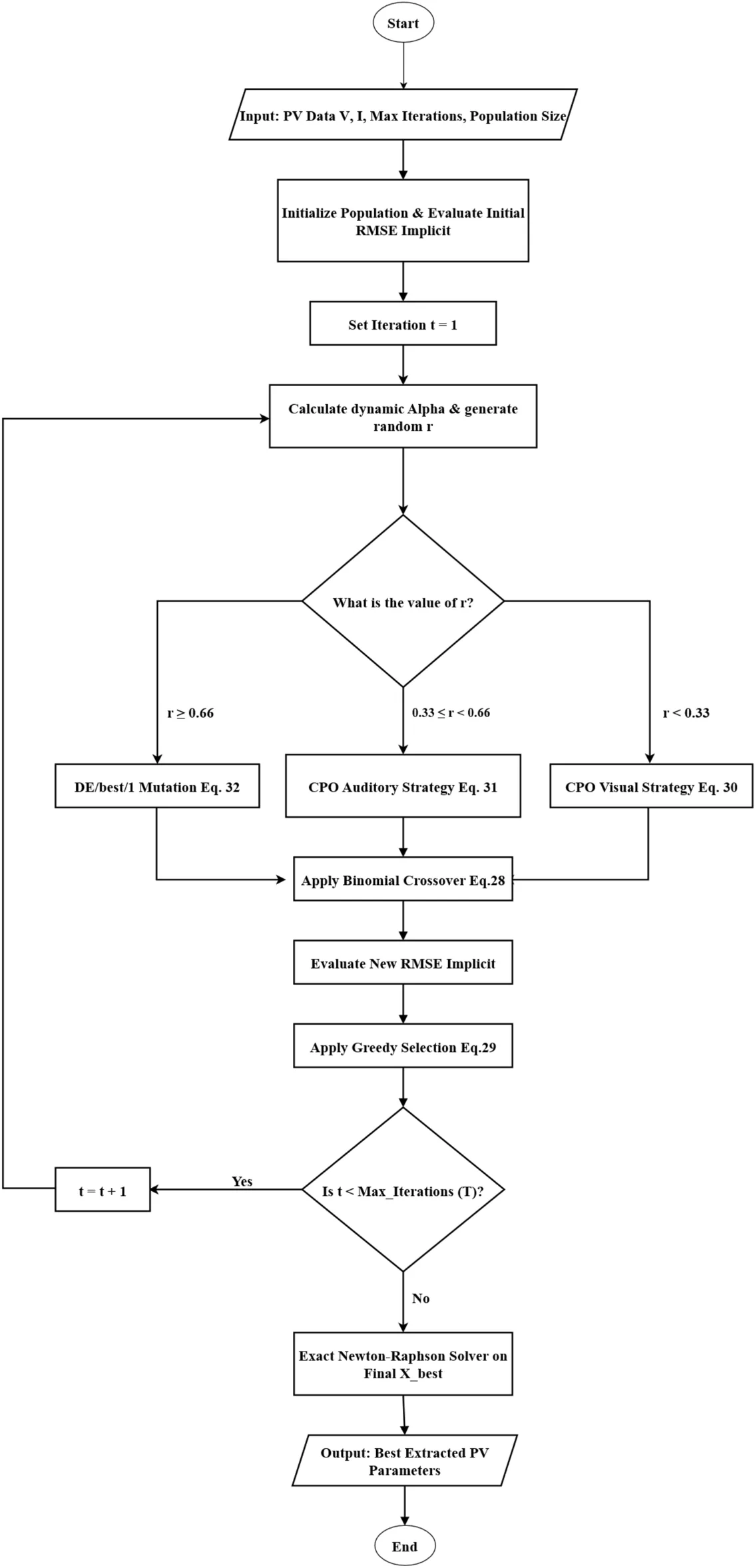
Flowchart of the proposed hybrid Crested Porcupine Differential Evolution (CPO-DE) algorithm.

The overall computational framework of the proposed CPO-DE algorithm is visually summarized in Fig. 4. The flowchart illustrates the sequence of the optimization process, highlighting the dynamic probability-based branching mechanism, the integration of the stability filter (binomial crossover), and the application of the multi-stage evaluation strategy. As depicted, the algorithm ensures that the computationally expensive Newton–Raphson explicit solver is executed exclusively at the final stage upon the global best solution. The detailed algorithmic steps, including the stochastic switching and parameter update rules, are formally presented in Algorithm 1.

Pseudocode of the proposed CPO-DE algorithm

**Algorithm 1:**
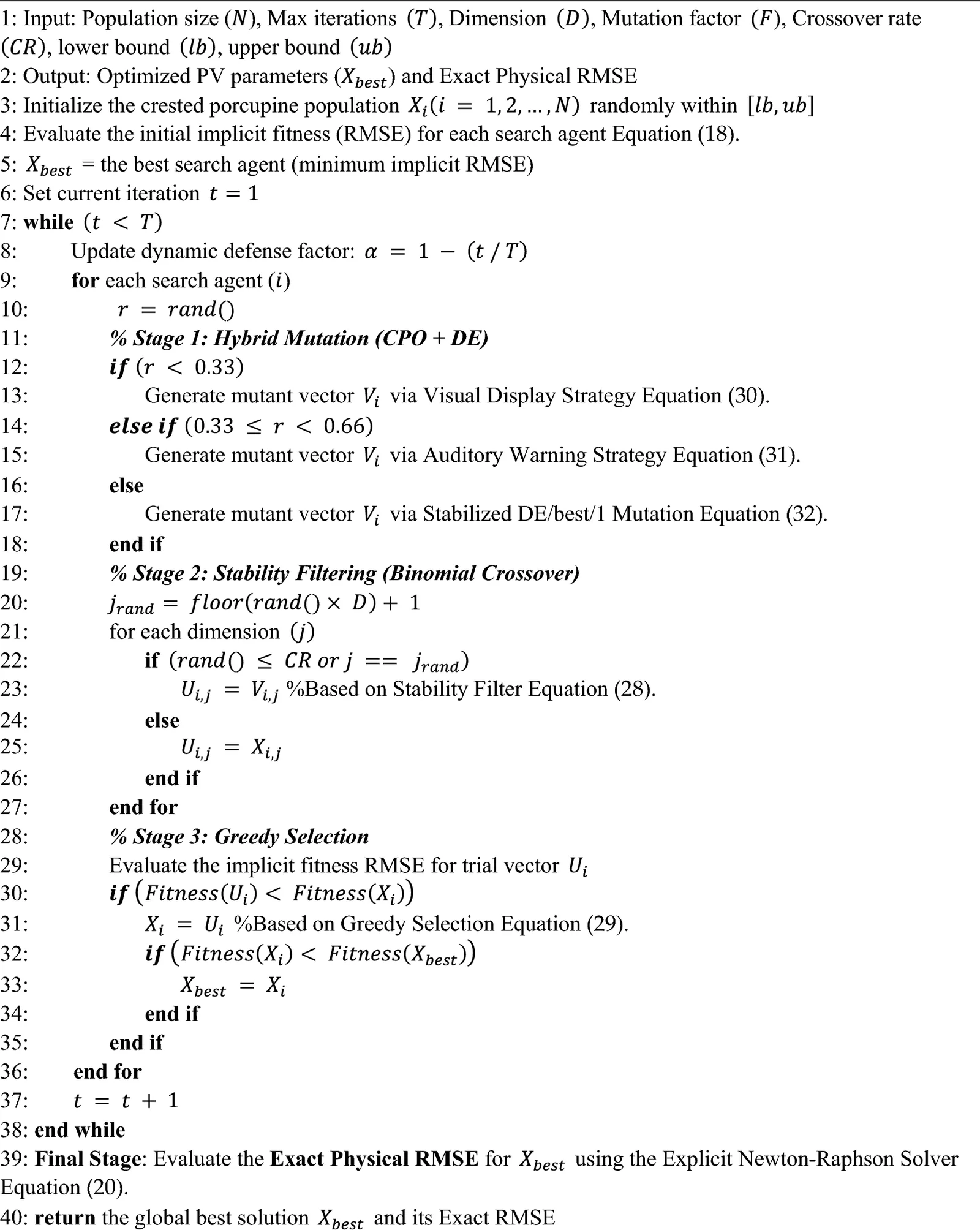
Proposed hybrid CPO-DE for PV parameter estimation.

#### Time complexity of CPO-DE

The computational time complexity of the proposed CPO-DE algorithm is a crucial factor in evaluating its efficiency. The complexity primarily depends on three phases: initialization, fitness evaluation, and position updating. Let *m* be the population size, *d* be the problem dimensionality, *T* be the maximum number of iterations, and *C* be the computational cost of evaluating the objective function.

The initialization phase requires $O\left(m\times d\right)$ operations. During the evolutionary loop, evaluating the fitness function for the entire population takes $O\left(T\times m\times C\right)$, while the position updating mechanism (including the hybrid CPO-DE mutation and crossover) requires $O\left(T\times m\times d\right)$ operations. Therefore, the overall time complexity is $O\left(m\times d+T\times m\times C+T\times m\times d\right)$, which simplifies to $O\left(T\times m\times \left(d+C\right)\right)$. This demonstrates that the hybridization strategy maintains the standard computational complexity of classical algorithms while significantly improving optimization robustness.

## Results and discussion

This section evaluates the proposed CPO-DE algorithm for optimal parameter estimation in solar PV models. The evaluation spans three levels of equivalent circuit complexity: SDM, DDM, and TDM. To verify its performance, the hybrid algorithm is benchmarked across standard mathematical functions, a constrained engineering design problem, and finally, the highly non-linear PV models, comparing it against a diverse set of classical and state-of-the-art metaheuristic techniques.

### Simulation and computational setup

Due to the inherent stochastic nature of meta-heuristic algorithms, drawing conclusions from a single execution is statistically inadequate. Therefore, to ensure a fair and robust comparison, all algorithms were evaluated under identical experimental conditions using a rigorous statistical framework.

All simulations and mathematical formulations were implemented and executed in MATLAB R2024b. The computational tasks were performed on a workstation equipped with an Intel Core™ Ultra 7 255HX processor (2.4 GHz, 20 cores) and 16 GB of RAM. Regarding the specific simulation environment for the PV parameter estimation phase, the objective function was evaluated using a sampling size of *N = 26* measured experimental data points obtained from the RTC France silicon solar cell. Furthermore, the final stage of the optimization framework invariably deploys the explicit Newton–Raphson numerical solver, configured with a rigorous convergence tolerance of $\epsilon ={10}^{-10}$ to guarantee exact physical precision. To guarantee an unbiased assessment, the unified control settings detailed in Table 2 were consistently applied across all twelve algorithms.

**Table 2:** The unified control settings applied to all algorithms.

Setting	Value
Population size	100 search agents
Maximum function evaluations (Max_FEs)	50,000 (which equals 500 iterations per run)
Independent runs	300 runs for each algorithm on each diode model

The parameter settings outlined in Table 2 were systematically selected to ensure a robust and fair evaluation. Because meta-heuristic performance is highly sensitive to these values, the population size and maximum iterations were empirically tuned. This specific configuration guarantees a sufficient number of function evaluations to reach the global optimum while avoiding unnecessary computational overhead. Furthermore, for the proposed CPO-DE algorithm, the scaling factor $\left(F\right)$ and crossover rate $\left(CR\right)$ were strictly adopted from established DE literature to maintain an optimal balance between broad exploration and deep exploitation. Beyond careful parameter tuning, assessing true algorithmic reliability requires rigorous statistical validation. Therefore, executing 300 independent runs was implemented as a standard procedure. This extensive replication provides a transparent and accurate assessment of algorithmic stability, effectively exposing any susceptibility to premature convergence especially as the dimensionality and complexity of the evaluated PV models increase.

### Statistical validation using benchmark functions

Before addressing the complex PV parameter estimation problem, we rigorously validated CPO-DE's mathematical robustness using 23 standard benchmark functions^46^ detailed in supplementary material. These functions evaluate different algorithmic capabilities. Specifically, unimodal functions (F1–F7) gauge convergence speed and pure exploitation. Multimodal functions (F8–F13) challenge exploration and the ability to escape local minima, while fixed-dimension multimodal functions (F14–F23) assess stability and search balance in restricted spaces^47^.

To ensure a fair and comprehensive evaluation, we selected two distinct groups of comparative algorithms. The first group includes the parent algorithms (CPO and DE) to explicitly verify that the hybridization process yielded measurable improvements over the standalone components. The second group comprises highly cited foundational optimizers: Particle Swarm Optimization (PSO)^48^, Genetic Algorithm (GA)^49^, Grey Wolf Optimizer (GWO)^50^, and Whale Optimization Algorithm (WOA)^51^ to benchmark CPO-DE against established industry standards. We evaluated the statistical significance of these comparisons using the Wilcoxon rank-sum test (5% significance level) across 50 independent runs. Table 3 details these quantitative results, and Table 4 summarizes the overall win/draw/loss ratios.

**Table 3:** Comprehensive benchmark results: mean, std dev and Wilcoxon test (50 runs).

Function	Metric	CPO-DE	CPO	DE	WOA	PSO	GWO	GA
F1	Mean	1.7815E−04	1.0788E+01 (+)	2.5541E−83 (=)	4.1787E−05 (=)	6.9095E−05 (=)	1.5035E−36 (=)	2.4228E+01 (+)
	Std Dev	1.1490E−03	2.9759E+01	1.7696E−82	3.5432E−05	6.3627E−05	2.6999E−36	1.0759E+01
F2	Mean	3.5486E−01	1.7664E+01 (+)	2.2410E−54 (−)	3.9037E−03 (−)	2.2638E−03 (−)	9.2452E−22 (−)	1.3701E+00 (+)
	Std Dev	1.0849E+00	2.0768E+01	8.2432E−54	1.6411E−03	4.4278E−03	8.7379E−22	3.8790E−01
F3	Mean	3.9411E+02	1.8898E+03 (+)	3.0553E+04 (+)	3.1135E+02 (=)	2.4668E+02 (−)	1.5687E−07 (−)	4.1802E+03 (+)
	Std Dev	3.0141E+02	1.5719E+03	1.1443E+04	1.3650E+02	1.0875E+02	4.7234E−07	1.8483E+03
F4	Mean	1.6325E+01	1.5463E+01 (=)	3.3877E+01 (+)	9.7015E+00 (−)	2.6952E+00 (−)	1.0763E−07 (−)	1.4076E+01 (−)
	Std Dev	4.1263E+00	4.1488E+00	2.8842E+01	4.7322E+00	6.3344E−01	1.5638E−07	2.3785E+00
F5	Mean	1.0890E+02	1.0574E+03 (+)	2.7324E+01 (−)	5.2873E+01 (−)	7.8236E+01 (=)	2.6717E+01 (−)	1.5810E+03 (+)
	Std Dev	1.0504E+02	2.5164E+03	3.9425E−01	6.5193E+01	8.8912E+01	7.0490E−01	9.8355E+02
F6	Mean	8.1178E−07	1.9273E+01 (=)	6.9631E−02 (+)	2.8848E−05 (+)	9.0981E−05 (+)	3.9670E−01 (+)	2.5874E+01 (+)
	Std Dev	3.7083E−06	8.0888E+01	5.7876E−02	2.3098E−05	8.7898E−05	3.6852E−01	1.5274E+01
F7	Mean	9.7342E−02	6.9036E−01 (+)	1.9234E−03 (−)	3.1354E−02 (−)	1.5657E−02 (−)	1.4592E−03 (−)	3.7319E−02 (−)
	Std Dev	6.6594E−02	3.5147E−01	2.2196E−03	9.7237E−03	5.4191E−03	7.0637E−04	1.4222E−02
F8	Mean	− 8.2651E+03	− 7.6240E+03 (+)	− 1.0883E+04 (−)	− 5.9887E+03 (+)	− 6.9112E+03 (+)	− 6.4902E+03 (+)	− 1.0161E+04 (−)
	Std Dev	6.4390E+02	7.7944E+02	1.6555E+03	8.4325E+02	6.9327E+02	1.2074E+03	6.9284E+02
F9	Mean	5.5757E+01	6.9602E+01 (+)	0.0000E+00 (−)	1.8935E+02 (+)	4.4137E+01 (−)	7.8530E+00 (−)	1.4501E+01 (−)
	Std Dev	1.5751E+01	2.6902E+01	0.0000E+00	1.5355E+01	1.3076E+01	7.1563E+00	3.2231E+00
F10	Mean	6.5531E+00	9.9403E+00 (+)	3.4994E−15 (−)	1.9175E−03 (−)	2.6966E−02 (−)	3.7961E−14 (−)	2.7697E+00 (−)
	Std Dev	1.4788E+00	2.1295E+00	2.2709E−15	1.0375E−03	1.6304E−01	4.4339E−15	3.9026E−01
F11	Mean	1.3020E−01	8.2267E−01 (+)	3.9034E−03 (−)	2.7416E−03 (−)	1.4728E−02 (−)	2.9936E−03 (−)	1.2617E+00 (+)
	Std Dev	1.7952E−01	6.4757E−01	1.9965E−02	6.5116E−03	1.7104E−02	5.9862E−03	1.3246E−01
F12	Mean	2.7763E+00	9.9602E+00 (+)	6.7378E−03 (−)	3.4676E−02 (−)	1.0370E−02 (−)	2.7363E−02 (−)	4.8123E−01 (−)
	Std Dev	2.5419E+00	5.9054E+00	4.5386E−03	9.7226E−02	3.1416E−02	1.4409E−02	3.8451E−01
F13	Mean	1.3635E+01	3.0491E+01 (+)	1.7902E−01 (−)	7.1392E−01 (−)	3.7695E−03 (−)	3.9329E−01 (−)	2.5050E+00 (−)
	Std Dev	8.7384E+00	9.4913E+00	1.3751E−01	1.6653E+00	9.3047E−03	1.9095E−01	1.3553E+00
F14	Mean	1.1367E+00	6.7440E+00 (+)	2.0597E+00 (+)	9.9800E−01 (=)	2.1844E+00 (+)	3.1191E+00 (+)	1.0105E+00 (=)
	Std Dev	6.0053E−01	5.5647E+00	2.4182E+00	0.0000E+00	1.7355E+00	3.2438E+00	8.8544E−02
F15	Mean	2.0659E−03	6.9106E−03 (+)	7.9873E−04 (=)	2.5195E−03 (=)	8.1612E−04 (=)	3.5390E−03 (=)	2.9882E−03 (=)
	Std Dev	5.4621E−03	1.1272E−02	8.1937E−04	6.0223E−03	2.8380E−03	7.4184E−03	5.8148E−03
F16	Mean	− 1.0316E+00	− 1.0316E+00 (=)	− 1.0316E+00 (+)	− 1.0316E+00 (=)	− 1.0316E+00 (=)	− 1.0316E+00 (+)	− 1.0316E+00 (=)
	Std Dev	2.3093E−16	4.0869E−16	4.1886E−10	2.2430E−16	2.4365E−16	7.0659E−09	2.3809E−05
F17	Mean	3.9789E−01	3.9789E−01 (=)	3.9789E−01 (+)	3.9789E−01 (=)	3.9789E−01 (=)	3.9789E−01 (=)	3.9799E−01 (=)
	Std Dev	0.0000E+00	0.0000E+00	4.1059E−06	0.0000E+00	0.0000E+00	3.0206E−05	3.8320E−04
F18	Mean	3.0000E+00	3.0000E+00 (+)	3.0000E+00 (=)	3.0000E+00 (−)	3.0000E+00 (+)	3.0000E+00 (+)	3.0001E+00 (+)
	Std Dev	1.0170E−15	4.3641E−15	7.2989E−05	1.1970E−15	1.2940E−15	1.1963E−05	1.2805E−04
F19	Mean	− 3.8628E+00	− 3.8628E+00 (=)	− 3.8585E+00 (+)	− 3.8628E+00 (=)	− 3.8628E+00 (=)	− 3.8617E+00 (+)	− 3.8628E+00 (+)
	Std Dev	1.3458E−15	9.8487E−16	5.8548E−03	1.3458E−15	1.3155E−15	2.3547E−03	2.9534E−06
F20	Mean	− 3.2649E+00	− 3.2602E+00 (=)	− 3.2406E+00 (=)	− 3.2388E+00 (+)	− 3.2839E+00 (=)	− 3.2607E+00 (=)	− 3.2958E+00 (−)
	Std Dev	6.0002E−02	6.0002E−02	9.1993E−02	5.5037E−02	5.6024E−02	7.2297E−02	4.9760E−02
F21	Mean	− 6.1943E+00	− 5.6427E+00 (=)	− 8.2715E+00 (−)	− 1.0052E+01 (−)	− 5.8971E+00 (=)	− 8.8943E+00 (−)	− 5.8128E+00 (=)
	Std Dev	3.5025E+00	3.2707E+00	2.6978E+00	7.1452E−01	3.3559E+00	2.4482E+00	3.7306E+00
F22	Mean	− 6.1784E+00	− 6.7628E+00 (=)	− 8.1306E+00 (−)	− 1.0403E+01 (−)	− 6.6256E+00 (=)	− 1.0297E+01 (−)	− 6.9450E+00 (=)
	Std Dev	3.5611E+00	3.6103E+00	3.1549E+00	1.3186E−15	3.6083E+00	7.4585E−01	3.6510E+00
F23	Mean	− 6.0608E+00	− 6.0016E+00 (=)	− 7.9487E+00 (−)	− 1.0249E+01 (−)	− 6.6446E+00 (=)	− 1.0049E+01 (−)	− 7.8480E+00 (−)
	Std Dev	3.6005E+00	3.8173E+00	3.2549E+00	1.4252E+00	3.8561E+00	1.9466E+00	3.6369E+00

**Table 4:** Statistical significance summary overview.

Algorithm	Wins (+)	Draws (=)	Losses (−)
CPO	14	9	0
DE	7	4	12
WOA	4	7	12
PSO	4	10	9
GWO	6	4	13
GA	8	6	9

The statistical summary highlights the direct success of the proposed hybridization. Evaluated against the original CPO, CPO-DE recorded 14 wins, 9 draws, and zero losses across the 23 test functions. This empirically proves that integrating DE operators effectively resolved the original CPO's stagnation issues, delivering better mean values and tighter standard deviations without sacrificing performance on any single function.

When compared to established baselines like GWO and DE, the results reflect a deliberate algorithmic trade-off that perfectly aligns with the No Free Lunch (NFL) theorem^37^. While CPO-DE recorded several losses against these algorithms, Table 3 shows that these losses were almost entirely confined to unimodal functions (F1–F7) which strictly test rapid exploitation. Conversely, CPO-DE demonstrated clear superiority in the highly multimodal landscapes of the multimodal functions (F8–F23).

This performance divergence is not a weakness, but a strategic design choice. The hybridization intentionally prioritizes deep exploration and local minima avoidance over pure exploitation speed. While this dynamic may slightly compromise performance in smooth, abstract mathematical functions, it provides the exact exploratory power required to solve highly non-linear, multi-dimensional engineering challenges like the Triple Diode Model (TDM).

#### Qualitative analysis (3D landscapes and convergence)

**Fig. 5:**
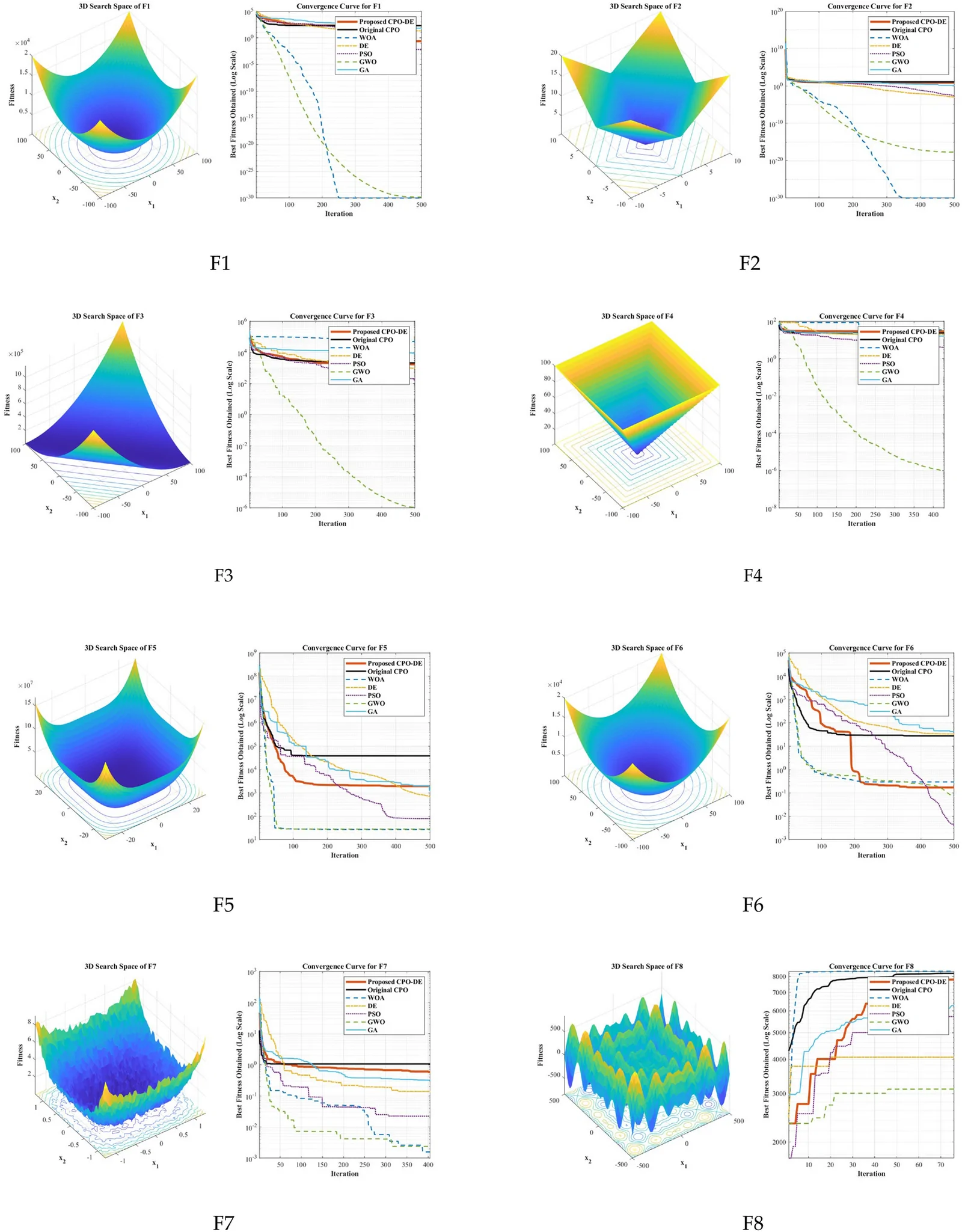

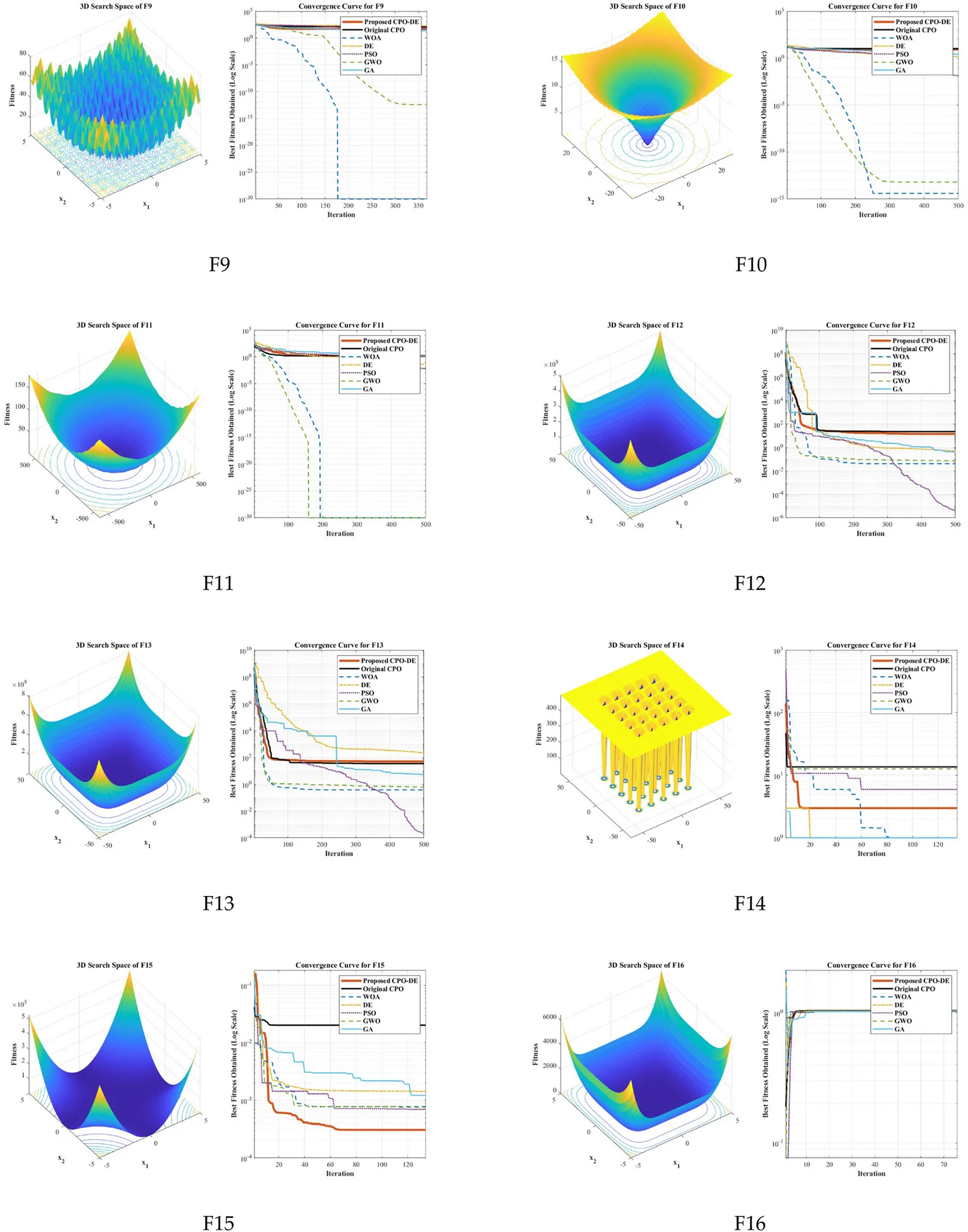

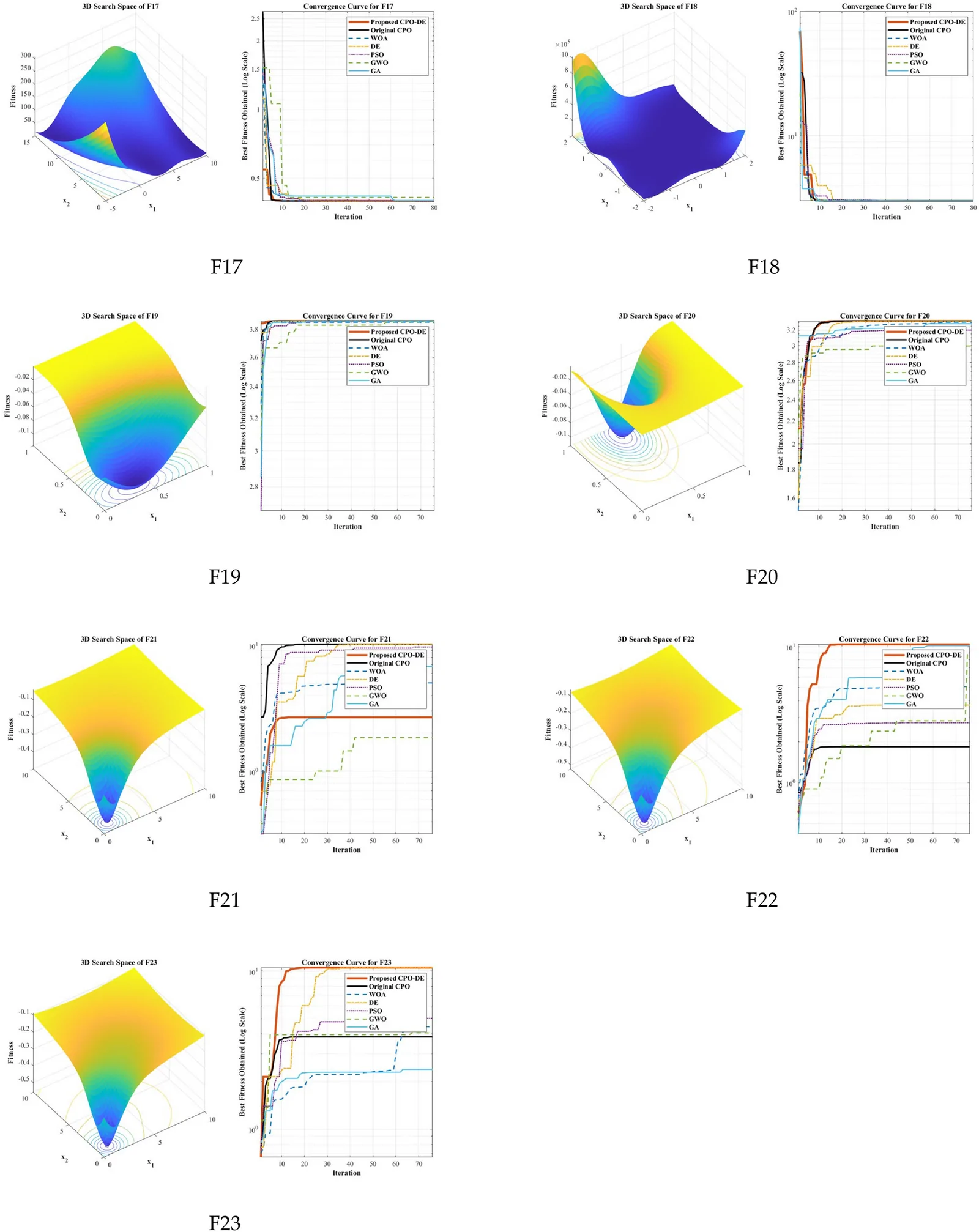
Search space topologies and convergence curves for the 23 standard benchmark functions (F1 to F23).

To complement the statistical analysis, Fig. 5 presents the 3D search landscapes and logarithmic convergence curves for all the 23 standard benchmark functions (F1–F23). The 3D topographies illustrate the severe complexity and deceptive local traps inherent in these problem spaces.

The convergence profiles visually confirm CPO-DE's superior search dynamics. The proposed algorithm initially exhibits a rapid descent, indicating robust exploration capabilities. More importantly, its true advantage emerges in the later iterations. Where the original CPO and competing algorithms prematurely stagnate, CPO-DE continuously refines its position, successfully escaping local minima to locate a substantially deeper global optimum. This graphical evidence directly corroborates the statistical data, proving that the integration of DE operators effectively resolves the early stagnation vulnerability of the standard CPO framework.

### Validation on classical engineering applications

To evaluate the robustness and constraint-handling capabilities of the proposed CPO-DE algorithm beyond solar PV applications, the framework was tested on three standard mechanical engineering benchmark problems^52^. The detailed mathematical formulations, objective functions, and penalty boundary conditions for these problems are provided in the Supplementary Material to maintain the manuscript's conciseness.

#### Tension/compression spring design problem

The objective of this problem is to minimize the weight of a spring subject to constraints on minimum deflection, shear stress, and surge frequency. The performance of the algorithms over 300 independent runs is detailed in Table 5.

**Table 5:** Results of the tension/compression spring design problem.

Algorithm	d(in)	D(in)	N(−)	Best	Worst	Mean	Std_Dev
CPO_DE	0.051689	0.356713	11.289217	1.266523E−2	1.270531E−2	1.266690E−2	3.235198E−6
CPO	0.051685	0.356628	11.294205	1.266523E−2	1.777316E−2	1.297518E−2	1.035353E−3
DE	0.051689	0.356718	11.288966	1.266523E−2	1.266523E−2	1.266523E−2	7.040772E−12
GWO	0.051565	0.353717	11.468838	1.266770E−2	1.309769E−2	1.272265E−2	4.634566E−5
GOA	0.051669	0.356229	11.317679	1.266524E−2	1.787306E−2	1.369813E−2	1.663143E−3

#### Welded beam design problem

This problem aims to minimize the fabrication cost of a welded beam under shear stress, bending stress, and buckling constraints. The statistical summary is presented in Table 6.

**Table 6:** Results of the welded beam design problem.

Algorithm	h(in)	l(in)	t(in)	b(in)	Best	Worst	Mean	Std_Dev
CPO_DE	0.205730	3.470489	9.036624	0.205730	1.724852E+0	1.724852E+0	1.724852E+0	1.362980E−15
CPO	0.205730	3.470489	9.036624	0.205730	1.724852E+0	2.002058E+0	1.726307E+0	1.767528E−2
DE	0.205730	3.470489	9.036624	0.205730	1.724852E+0	1.724852E+0	1.724852E+0	3.853562E−14
GWO	0.205658	3.472176	9.037480	0.205744	1.725230E+0	1.732898E+0	1.726718E+0	1.198795E−3
GOA	0.205756	3.470202	9.036063	0.205756	1.724958E+0	2.376729E+0	1.805576E+0	1.137462E−1

#### Pressure vessel design problem

The objective is to minimize the total cost of material, forming, and welding of a cylindrical vessel. The results are shown in Table 7.

**Table 7:** Results of the pressure vessel design problem.

Algorithm	Ts(in)	Th(in)	R(in)	L(in)	Best	Worst	Mean	Std_Dev
CPO_DE	0.778169	0.384649	40.319619	200.000000	5.885333E+3	5.887780E+3	5.885378E+3	2.491830E−1
CPO	0.778169	0.384649	40.319619	200.000000	5.885333E+3	7.319001E+3	6.094920E+3	3.382377E+2
DE	0.778169	0.384649	40.319619	200.000000	5.885333E+3	5.885333E+3	5.885333E+3	3.028119E−11
GWO	0.778585	0.384931	40.338823	199.737257	5.886676E+3	7.289631E+3	5.967226E+3	2.354577E+2
GOA	0.778170	0.384654	40.319675	200.000000	5.885364E+3	2.001029E+5	7.009650E+3	1.119441E+4

#### Discussion of engineering benchmarks

A comprehensive analysis of Tables 5, 6, and 7 reveals a consistent behavioral pattern among the tested algorithms across the three constrained engineering problems. The standard CPO demonstrates strong global exploration capabilities, frequently locating the absolute minimum cost; however, it struggles with structural stability in highly constrained spaces, which is evident from its elevated standard deviations (e.g., $3.38\times {10}^{2}$ in the pressure vessel problem). Conversely, standard DE shows characteristic stability and precision in these continuous constrained environments, although it typically exhibits vulnerability to stagnation in the highly deceptive, multi-modal PV parameter extraction spaces evaluated earlier in this study. Meanwhile, GWO and GOA either converged prematurely to local optima or suffered from severe constraint violations, as demonstrated by GOA's inflated worst-cost in the pressure vessel problem.

The empirical outcomes of these benchmarks provide a clear, objective justification for the proposed hybridization. By integrating DE's mutation and crossover mechanisms, CPO-DE effectively mitigates the inherent boundary-handling instability of standard CPO, reducing its variance significantly without compromising its exploratory reach. Consequently, the CPO-DE architecture is validated as a well-balanced, general-purpose optimizer capable of navigating both the highly multi-modal landscapes of PV systems and the rigid constraints of classical mechanical design.

### Application to PV parameter estimation

Having established its mathematical robustness, we now apply the CPO-DE algorithm to our primary engineering challenge: estimating the unknown parameters of solar photovoltaic (PV) models. We evaluate the algorithm's performance across three progressively complex equivalent circuits. These are (SDM), (DDM), and the highly non-linear (TDM).

To guarantee a rigorous and unbiased assessment, we benchmarked CPO-DE against eleven diverse optimization algorithms. This comparative ensemble naturally includes the algorithm's parent components the CPO^38^ and DE^39^ to verify the hybridization success. We also tested against widely recognized foundational methods, namely the GA^49^, PSO^48^, GWO^50^, and WOA^51^. Finally, to ensure our proposed method holds up against recent literature, we incorporated state-of-the-art models published between 2022 and 2024. These feature the White Shark Optimizer (WSO)^53^, Snow Ablation Optimizer (SAO)^54^, Hippopotamus Optimization Algorithm (HOA)^55^, Gazelle Optimization Algorithm (GOA)^56^, and Dual-Population Gaining-Sharing Knowledge (DPGSK)^57^.

#### Case Study 1: Single Diode Model (SDM)

In this case study, all twelve algorithms were evaluated using experimental data from the RTC France silicon solar cell (tested at 33 °C and 1000 W/m^2^) to minimize the Root Mean Square Error (RMSE). Table 8 presents the estimated parameters, while Table 9 details the descriptive statistics over multiple independent runs.

**Table 8:** Comparative analysis of various parameter estimation techniques for the SDM.

Algorithm	${I}_{ph} \left(A\right)$	${I}_{sd}\left(A\right)$	${R}_{s} \left(\Omega \right)$	${R}_{sh} \left(\Omega \right)$	*n*
CPO_DE	0.760776	3.230208E−7	0.036377	53.718518	1.481718
CPO	0.760770	3.127275E−7	0.036507	52.884495	1.478466
DE	0.760772	3.225830E−7	0.036380	53.673903	1.481583
PSO	0.760797	3.083429E−7	0.036565	52.505635	1.477048
GWO	0.760842	2.696447E−7	0.037078	48.291869	1.463754
DPGSK	0.760776	3.230208E−7	0.036377	53.718520	1.481718
SAO	0.760836	3.172002E−7	0.036446	52.891297	1.479890
HOA	0.760724	3.099978E−7	0.036563	53.486726	1.477569
GOA	0.760159	2.368612E−7	0.037674	54.537160	1.450961
WOA	0.760154	3.462253E−7	0.036269	67.571706	1.488546
GA	0.760464	3.825301E−7	0.035834	63.415305	1.498841
WSO	0.760783	3.109100E−7	0.036524	52.590967	1.477886

**Table 9:** Statistical error analysis (RMSE) of various optimization techniques applied to the SDM.

Algorithm	Best	Worst	Mean	STDEV
CPO_DE	7.753913E−4	7.753913E−4	7.753913E−4	8.104005E−12
CPO	7.733679E−4	3.815132E−2	1.158196E−3	2.180715E−3
DE	7.752442E−4	1.022813E−3	7.979340E−4	2.967382E−5
PSO	7.732890E−4	2.121012E−3	1.059754E−3	2.345656E−4
GWO	8.199953E−4	4.535970E−2	5.875075E−3	9.977528E−3
DPGSK	7.753913E−4	7.753913E−4	7.753913E−4	6.750418E−12
SAO	7.742803E−4	7.544182E−3	1.708789E−3	1.074197E−3
HOA	7.739164E−4	1.026142E−2	2.128659E−3	1.210466E−3
GOA	9.954154E−4	2.200531E−2	8.269138E−3	7.582264E−3
WOA	9.291412E−4	4.975454E−2	1.129295E−2	1.597136E−2
GA	8.814656E−4	2.466141E−3	1.711560E−3	3.049806E−4
WSO	7.735314E−4	1.310441E−3	8.662722E−4	1.514682E−4

**Fig. 6:**
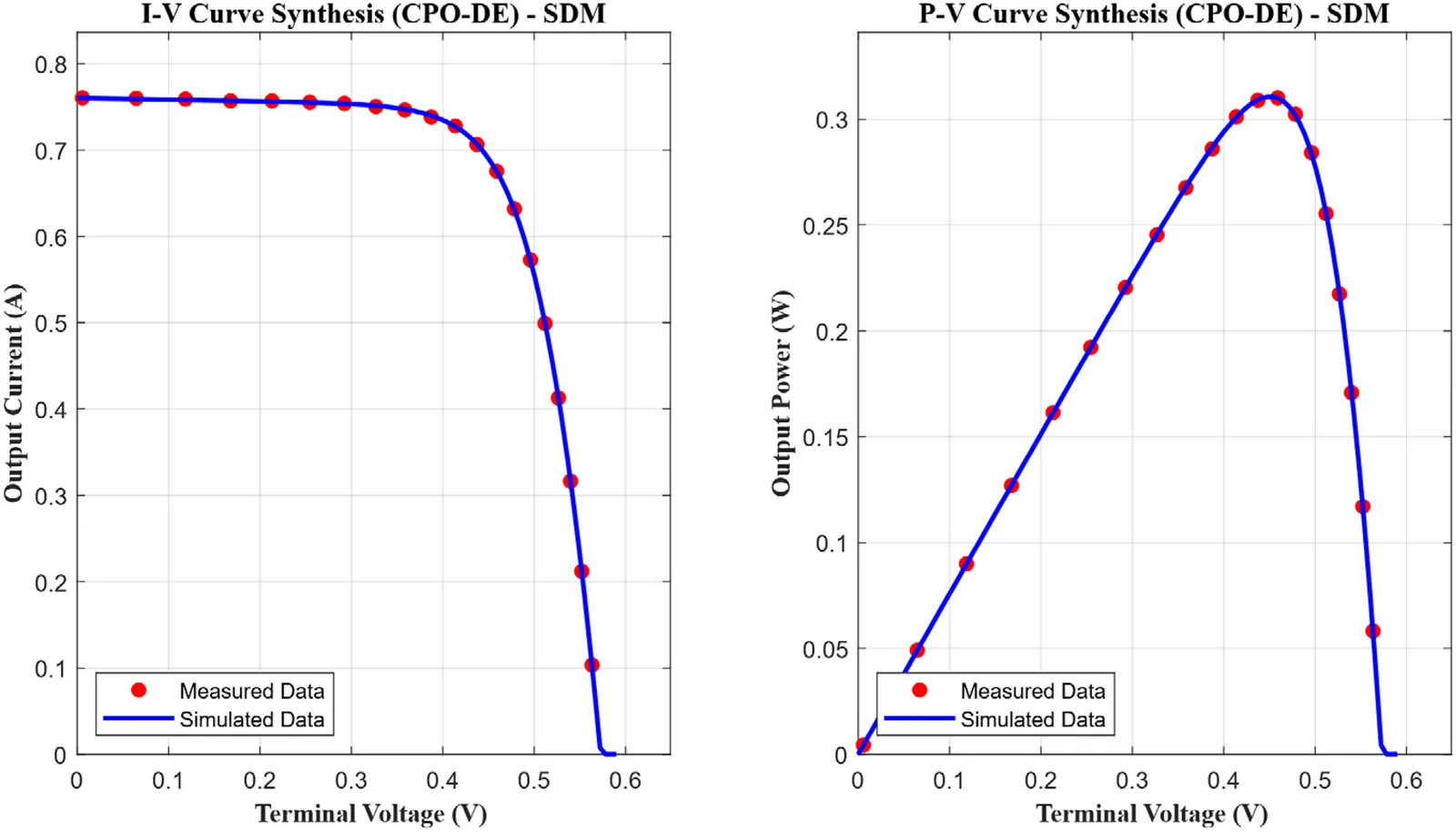
Graphical representation of the current–voltage and power-voltage characteristics for the SDM.

As observed in Table 9, while some algorithms such as the original CPO, PSO, and WSO achieved slightly lower absolute Best RMSE values in isolated runs, evaluating the true superiority of stochastic metaheuristics relies fundamentally on statistical robustness rather than a single best occurrence. The original CPO and PSO suffer from instability and high variance, as evidenced by their significantly higher Worst and Mean RMSE values, indicating occasional entrapment in local optima. In stark contrast, the proposed CPO-DE demonstrates superior overall performance by entirely eliminating this instability; it consistently converges to a highly competitive RMSE of $7.753913\times {10}^{-4}$ across all independent runs. The physical accuracy of the parameters estimated by CPO-DE is visually confirmed in Fig. 6, where the simulated I-V and P–V characteristic curves perfectly align with the actual measured data points of the RTC France solar cell.

**Fig. 7:**
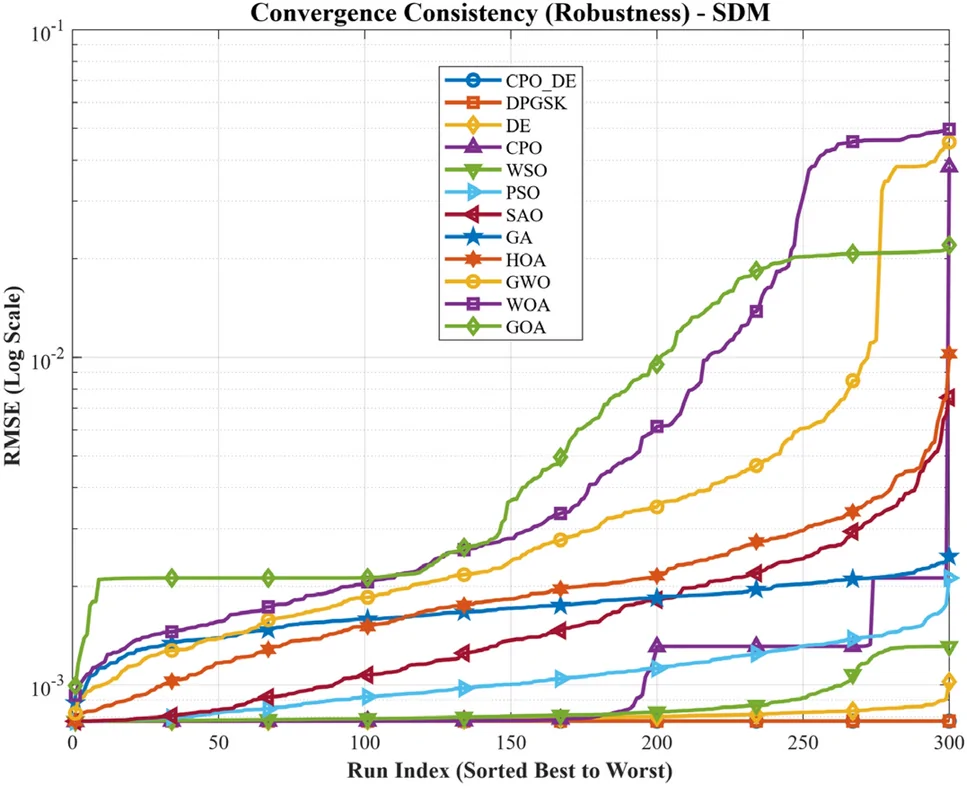
Stability and robustness of the evaluated algorithms for the SDM.

**Fig. 8:**
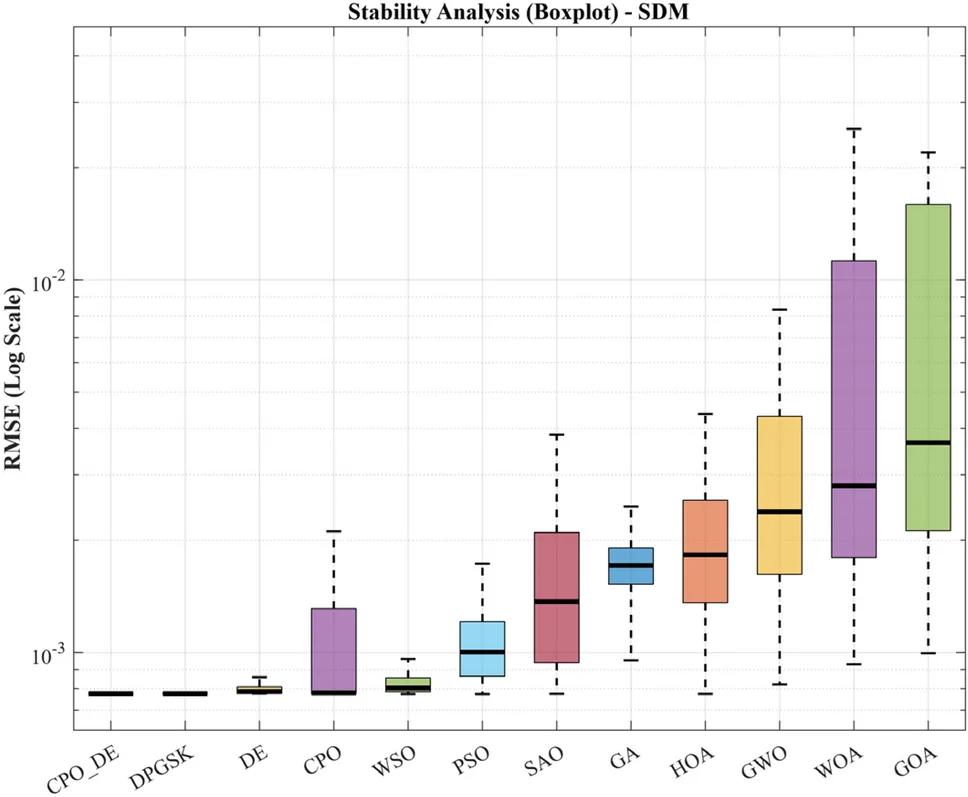
Stability analysis (Boxplot) for the SDM.

**Fig. 9:**
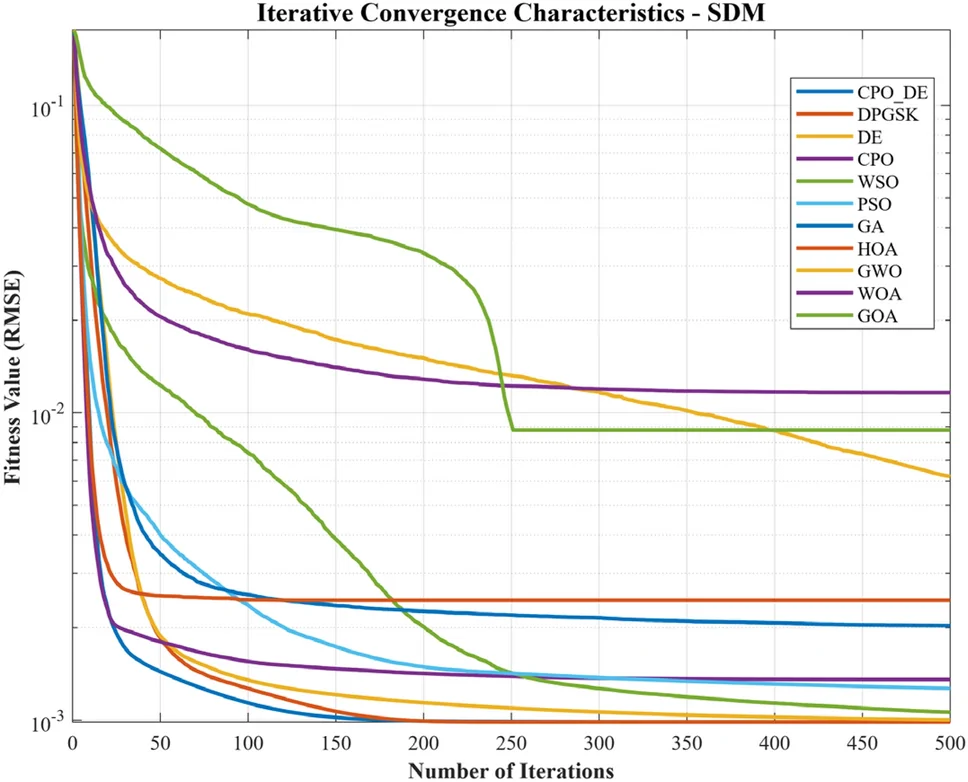
Iterative convergence characteristics for the SDM.

The primary contribution of CPO-DE lies in its high consistency and robustness. As highlighted in Table 6, CPO-DE achieved an exceptionally low standard deviation of $8.104\times {10}^{-12}$, with identically matching Best, Worst, and Mean RMSE values across all runs. This proves the hybridization strategy mitigated the original CPO's tendency to stagnate in local optima. This minimal variance and extreme stability are clearly depicted in the flat, horizontal trajectory of the robustness curve Fig. 7 and the compact boxplot Fig. 8. Furthermore, the algorithm's rapid computational efficiency and steep error descent are demonstrated in the iterative convergence curve Fig. 9.

**Fig. 10:**
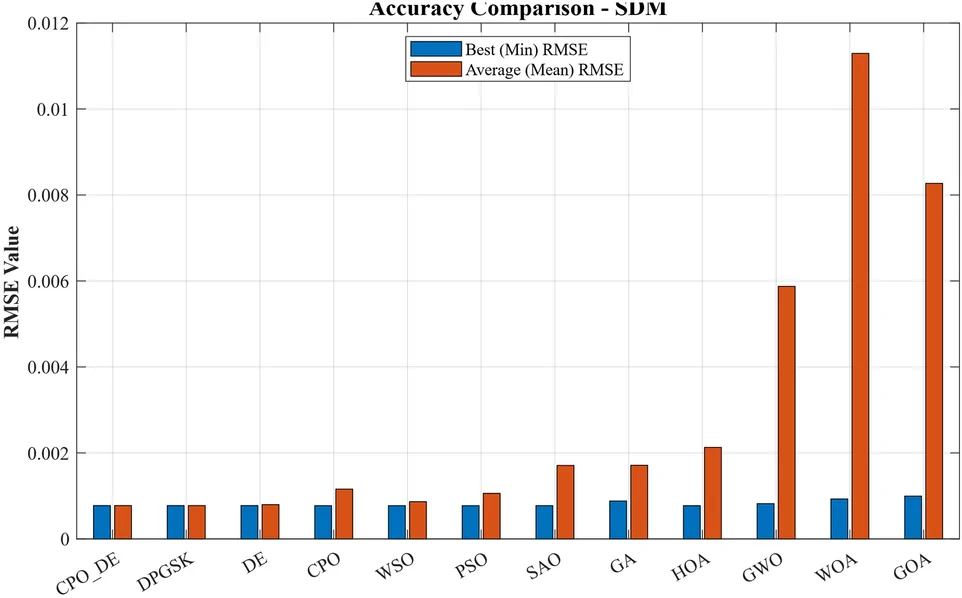
Accuracy comparison for the SDM.

Non-parametric tests rigorously validate these findings. The Wilcoxon rank-sum test Table 10 confirms that CPO-DE statistically outperforms 10 of the 11 algorithms, tying only with DPGSK. Ultimately, the Friedman mean rank test Table 11 ranks CPO-DE first with a Score of 1.7900. It is important to emphasize that the Friedman test calculates the average rank across all independent runs, not just the single best occurrence. Because algorithms like CPO and PSO suffer from high variance, they rank poorly in the majority of runs despite occasional optimums. Conversely, CPO-DE's near-zero variance ensures it maintains a top-tier rank in every single trial, firmly establishing it mathematically and statistically as the most reliable optimizer for the SDM. Figure 10 highlights the accuracy comparison, confirming the minimal gap between the best and mean values for the proposed algorithm.

**Table 10:** Wilcoxon rank-sum test (compared to baseline: CPO-DE) for the SDM.

Algorithm	p_value	Decision
CPO_DE	1.00000E+00	–
DPGSK	8.27873E−2	Draw (=)
DE	6.63504E−69	Loss (−)
CPO	1.81795E−44	Loss (−)
WSO	1.46636E−94	Loss (−)
PSO	8.59574E−91	Loss (−)
SAO	4.86318E−92	Loss (−)
GA	1.04970E−99	Loss (−)
HOA	2.09092E−98	Loss (−)
GWO	1.04970E−99	Loss (−)
WOA	1.04970E−99	Loss (−)
GOA	1.04970E−99	Loss (−)

**Table 11:** Friedman mean rank test (lower is better) for the SDM.

Final position	Algorithm	Mean Rank
Rank 1	CPO_DE	1.7900
Rank 2	DPGSK	1.8533
Rank 3	DE	3.8100
Rank 4	CPO	4.5333
Rank 5	WSO	4.6600
Rank 6	PSO	5.8233
Rank 7	SAO	7.5167
Rank 8	GA	8.4300
Rank 9	HOA	8.7067
Rank 10	GWO	9.7367
Rank 11	WOA	10.2333
Rank 12	GOA	10.9067

#### Case study 2: Double Diode Model (DDM)

**Fig. 11:**
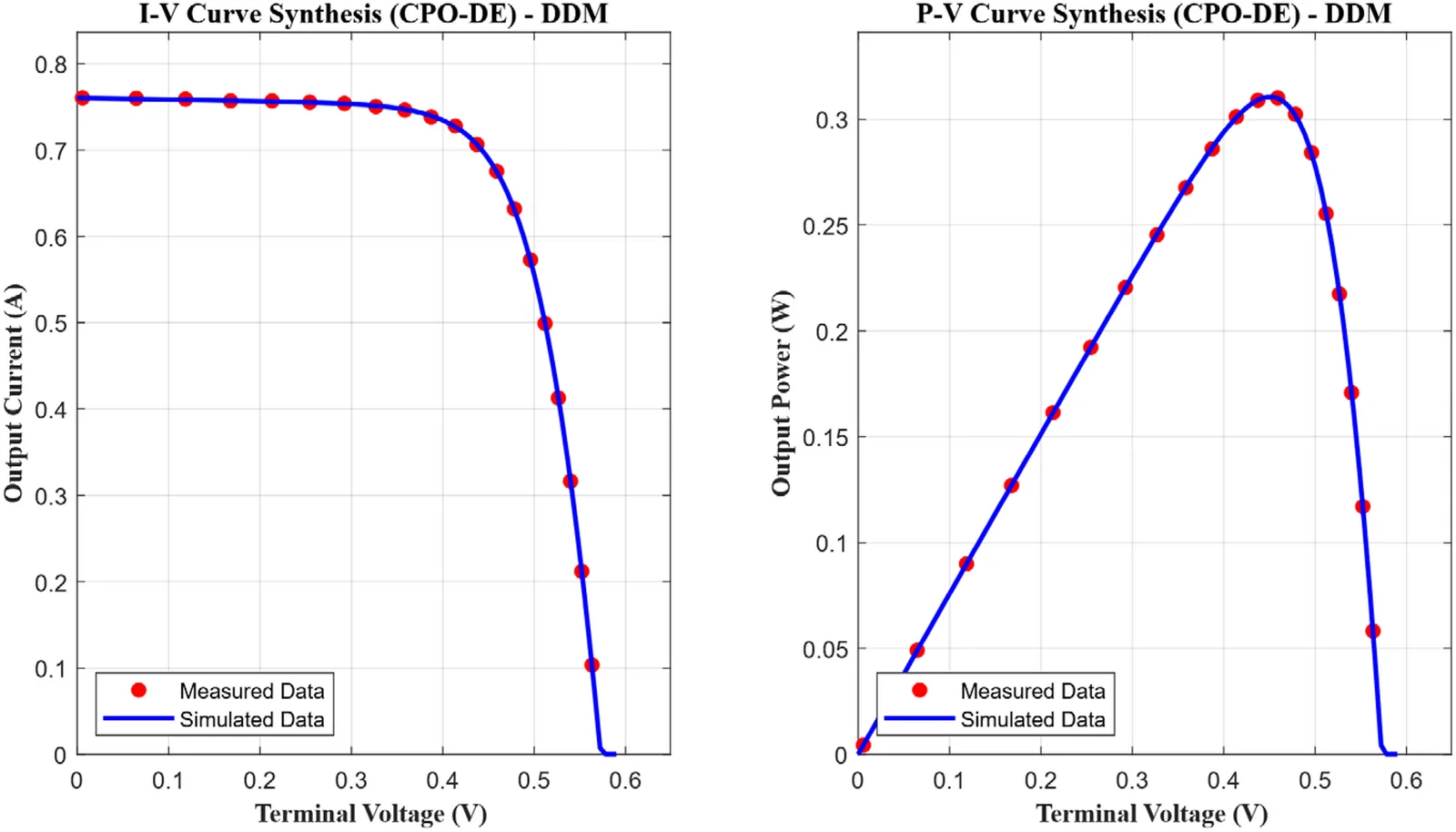
Graphical representation of the current–voltage and power-voltage characteristics for the DDM.

Transitioning to the higher-dimensional Double Diode Model (DDM), Table 12 details the optimal seven parameters estimated, while Table 13 provides the comparative statistical breakdown. In this highly non-linear search space, the proposed CPO-DE achieved a superior Best RMSE of $7.4789\times {10}^{-4}$, successfully outperforming highly competitive algorithms such as WSO and the original CPO. The physical validity of these parameters is visually confirmed by the accurately aligned I-V and P–V curves in Fig. 11.

**Table 12:** Comparative analysis of various parameter estimation techniques for the DDM.

Algorithm	${I}_{ph} \left(A\right)$	${I}_{sd1}\left(A\right)$	${R}_{s} \left(\Omega \right)$	${R}_{sh} \left(\Omega \right)$	${n}_{1}$	${I}_{sd2}\left(A\right)$	${n}_{2}$
CPO_DE	0.760784	9.134722E−8	0.037352	57.171265	1.384371	9.956463E−7	1.815320
CPO	0.760783	1.406074E−7	0.037102	56.624979	1.414882	1.000000E−6	1.889345
DE	0.760769	3.804694E−7	0.036561	54.772361	1.963868	2.644628E−7	1.464970
PSO	0.760934	7.929922E−8	0.037663	53.636331	1.375625	7.172374E−7	1.746634
GWO	0.760669	2.430669E−7	0.036823	53.251576	1.458327	1.813778E−7	1.780601
DPGSK	0.760786	4.369662E−7	0.036731	55.504143	1.815627	2.028638E−7	1.445223
SAO	0.760793	5.840367E−7	0.037053	55.323528	1.717800	1.024316E−7	1.398397
HOA	0.760908	1.682558E−7	0.037798	50.340127	1.424311	6.675768E−7	1.954329
GOA	0.760542	2.353886E−7	0.036437	73.796089	1.457081	1.000000E−6	2.000000
WOA	0.760279	2.255957E−7	0.035402	62.858910	1.999919	3.826636E−7	1.500625
GA	0.760604	6.137937E−8	0.033017	80.325648	1.876997	6.794545E−7	1.561717
WSO	0.760789	9.973621E−7	0.037096	56.755902	1.889944	1.414935E−7	1.415369

**Table 13:** Summary of root mean square error (RMSE) results obtained by competing algorithms for the DDM.

Algorithm	Best	Worst	Mean	STDEV
CPO_DE	7.478997E−4	7.951519E−4	7.666914E−4	6.701695E−6
CPO	7.497123E−4	2.715746E−3	1.079495E−3	4.119847E−4
DE	7.658155E−4	1.550562E−3	7.780000E−4	5.052798E−5
PSO	7.532473E−4	2.201491E−3	1.197259E−3	2.972711E−4
GWO	7.717096E−4	4.486898E−2	5.322628E−3	8.980856E−3
DPGSK	7.625769E−4	1.494319E−3	8.546015E−4	1.164054E−4
SAO	7.550158E−4	7.717678E−3	1.899091E−3	1.088540E−3
HOA	8.060097E−4	6.552524E−3	2.611258E−3	1.022308E−3
GOA	9.972980E−4	2.685821E−2	8.054233E−3	7.138234E−3
WOA	9.584807E−4	6.243135E−2	1.264596E−2	1.602097E−2
GA	1.555943E−3	4.054568E−3	2.535897E−3	4.219201E−4
WSO	7.497790E−4	1.456103E−3	9.736678E−4	1.990924E−4

**Fig. 12:**
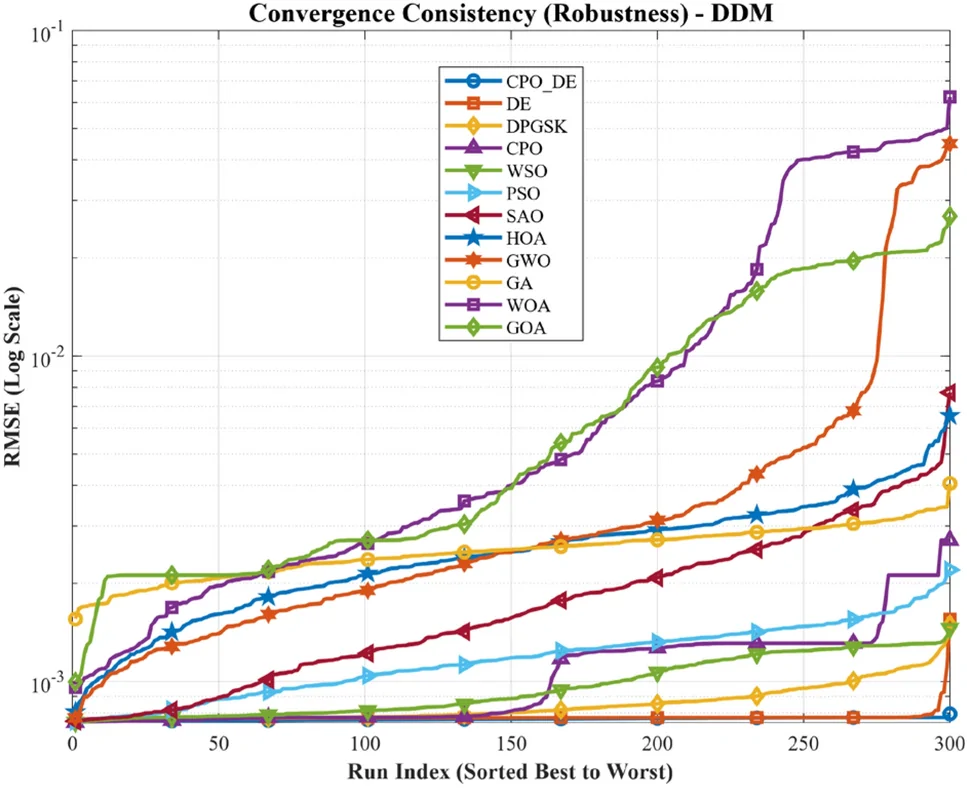
Stability and robustness of the evaluated algorithms for the DDM.

**Fig. 13:**
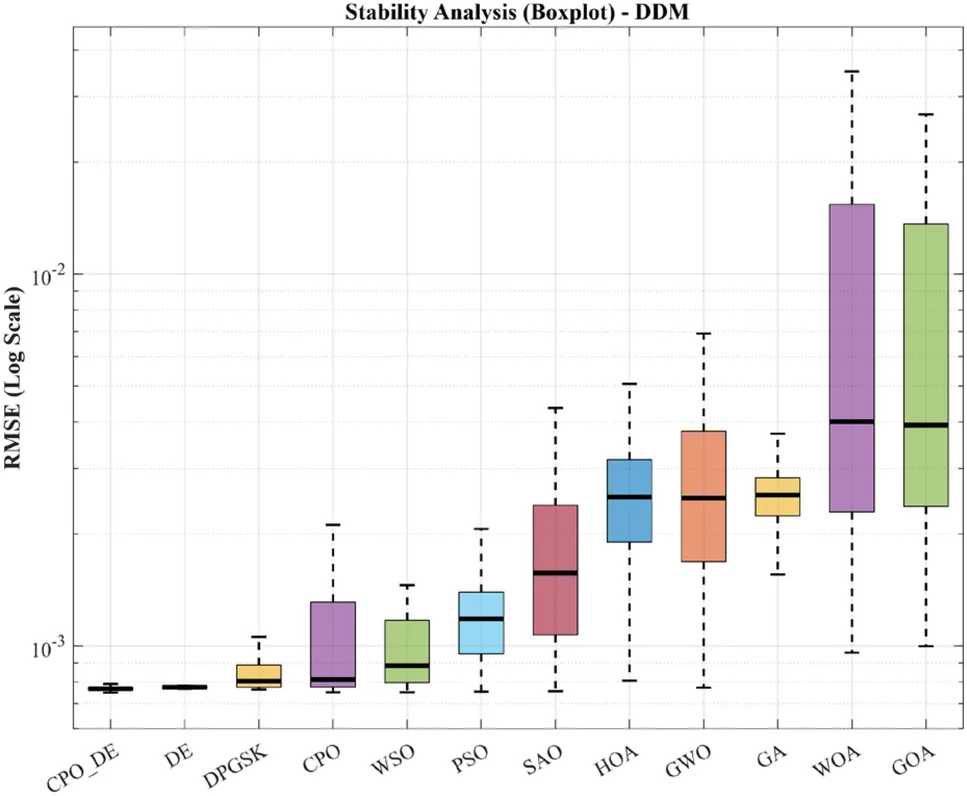
Stability analysis (Boxplot) for the DDM.

**Fig. 14:**
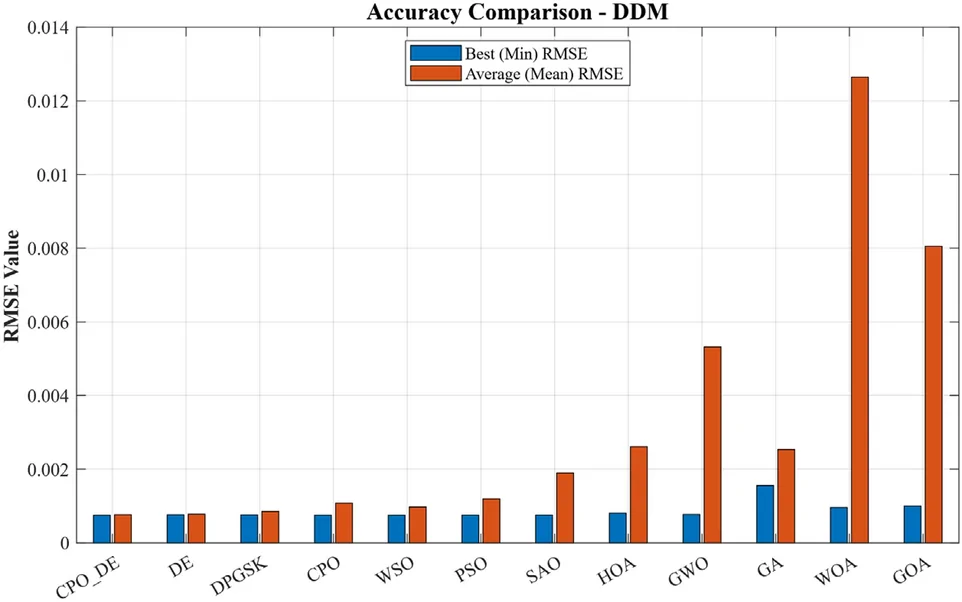
Accuracy comparison for the DDM.

**Fig. 15:**
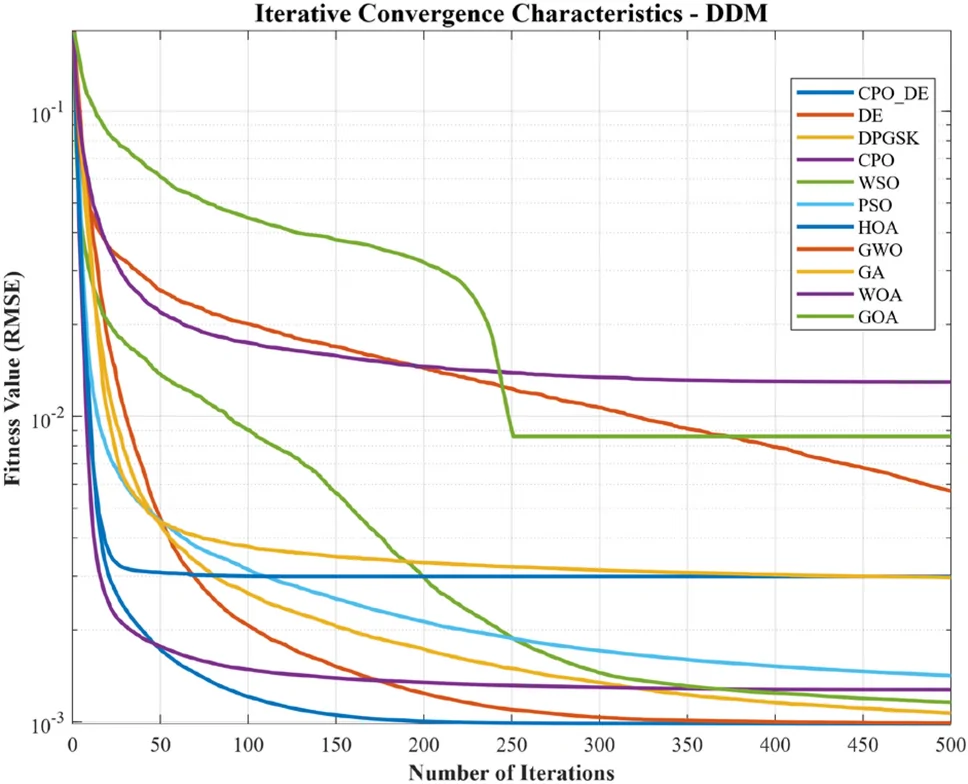
Iterative Convergence Characteristics for the DDM.

Beyond peak accuracy, CPO-DE demonstrated high robustness, recording a low standard deviation of $6.7016\times {10}^{-6}$. While algorithms like the original CPO and PSO suffered severe degradation in their worst runs due to local optima entrapment, CPO-DE maintained minimal variance. This stability across independent runs is clearly confirmed by the robustness curve Fig. 12 , the compact distribution in the boxplot Fig. 13 and the narrow best-to-mean RMSE gap Fig. 14. Furthermore, to evaluate the dynamic search behavior, the iterative convergence curve Fig. 15 confirms that the DE-based mutation strategy effectively prevented premature convergence across iterations.

Statistically, CPO-DE established strong performance in the DDM case. The Wilcoxon rank-sum test Table 14 indicates that CPO-DE significantly outperformed all 11 competing algorithms. Consequently, the Friedman mean rank test Table 15 secures CPO-DE in the 1st Rank with a mean score of 1.5933, cementing its superior capability in handling complex, multi-modal PV parameter estimation.

**Table 14:** Wilcoxon rank-sum test (compared to baseline: CPO-DE) for the DDM.

Algorithm	p_value	Decision
CPO_DE	1.00000E+00	–
DE	2.61945E−31	Loss (−)
DPGSK	4.40348E−68	Loss (−)
CPO	9.59224E−46	Loss (−)
WSO	1.34632E−80	Loss (−)
PSO	1.41076E−91	Loss (−)
SAO	1.20907E−89	Loss (−)
HOA	1.04970E−99	Loss (−)
GWO	2.50464E−99	Loss (−)
GA	1.04970E−99	Loss (−)
WOA	1.04970E−99	Loss (−)
GOA	1.04970E−99	Loss (−)

**Table 15:** Friedman mean rank test (lower is better) for the DDM.

Final position	Algorithm	Mean Rank
Rank 1	CPO_DE	1.5933
Rank 2	DE	2.4133
Rank 3	DPGSK	3.7233
Rank 4	CPO	4.3967
Rank 5	WSO	4.6000
Rank 6	PSO	5.7800
Rank 7	SAO	7.2267
Rank 8	HOA	9.0133
Rank 9	GWO	9.1267
Rank 10	GA	9.3467
Rank 11	WOA	10.3000
Rank 12	GOA	10.4800

#### Case Study 3: Triple Diode Model (TDM)

**Fig. 16:**
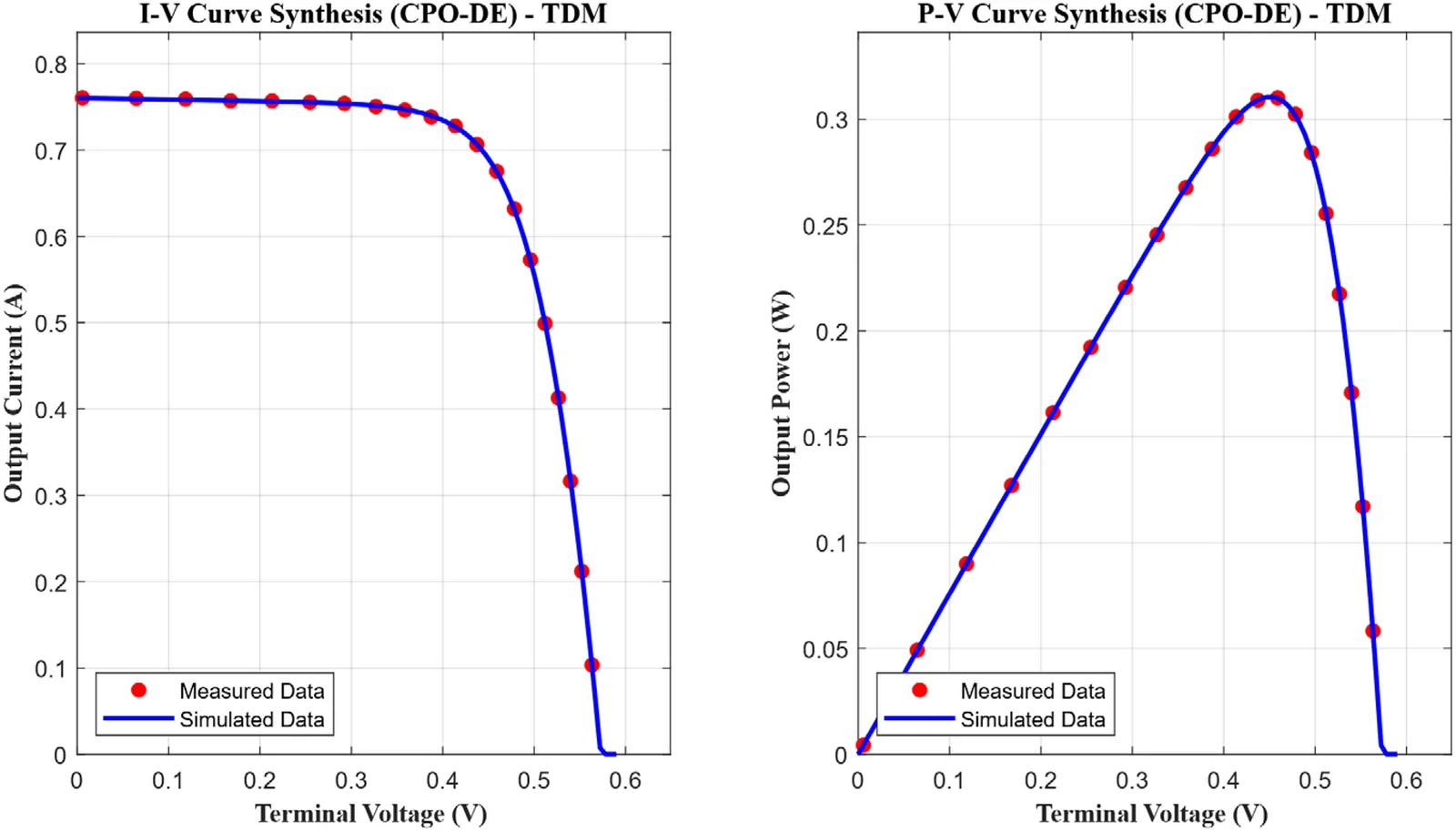
Graphical representation of the current–voltage and power-voltage characteristics for the TDM.

The Triple Diode Model (TDM) presents a highly complex, 9-dimensional optimization challenge. Table 16 details the estimated parameters, while Table 17 provides the comparative statistical evaluation. The proposed CPO-DE achieved a highly competitive Best RMSE of $7.4742\times {10}^{-4}$. While algorithms like the original CPO and SAO occasionally recorded slightly lower peak values, they suffered from high error rates in their worst runs. In contrast, CPO-DE guarantees reliable and physically accurate parameter estimation across all runs, as visually validated by the I-V and P–V curves in Fig. 16.

**Table 16:** Comparative analysis of various parameter estimation techniques for the TDM.

**Algorithm**	${{\boldsymbol{I}}}_{{\boldsymbol{p}}{\boldsymbol{h}}}\left({\boldsymbol{A}}\right)$	${{\boldsymbol{I}}}_{{\boldsymbol{s}}{\boldsymbol{d}}1}\left({\boldsymbol{A}}\right)$	${{\boldsymbol{R}}}_{{\boldsymbol{s}}}\left({\boldsymbol{\Omega}}\right)$	${{\boldsymbol{R}}}_{{\boldsymbol{s}}{\boldsymbol{h}}}\left({\boldsymbol{\Omega}}\right)$	${{\boldsymbol{n}}}_{1}$	${{\boldsymbol{I}}}_{{\boldsymbol{s}}{\boldsymbol{d}}2}\left({\boldsymbol{A}}\right)$	${{\boldsymbol{n}}}_{2}$	${{\boldsymbol{I}}}_{{\boldsymbol{s}}{\boldsymbol{d}}3}\left({\boldsymbol{A}}\right)$	${{\boldsymbol{n}}}_{3}$
CPO_DE	0.760771	3.087344E−7	0.037237	57.542913	1.976738	1.221670E−7	1.403844	8.732028E−7	1.876124
CPO	0.760851	1.228628E−7	0.037440	57.225057	1.401748	9.288175E−7	1.977710	7.324300E−7	2.000000
DE	0.760738	2.401262E−7	0.036750	55.768973	1.951278	2.318377E−7	1.453855	3.815918E−7	1.987626
PSO	0.760755	1.053857E−7	0.037029	57.724457	1.398084	3.546970E−7	2.000000	5.564976E−7	1.742266
GWO	0.761161	1.982528E−9	0.037189	48.929884	1.965562	2.355142E−7	1.453729	3.073317E−7	1.923551
DPGSK	0.760852	1.996709E−7	0.036790	55.410831	1.442591	3.825132E−7	1.934981	3.033638E−7	1.878489
SAO	0.760870	3.081381E−7	0.036911	53.966628	1.937935	3.812098E−7	1.849105	1.815550E−7	1.434982
HOA	0.759849	1.497608E−7	0.036500	67.083303	1.439290	8.830115E−8	1.920058	2.367330E−7	1.586718
GOA	0.761482	7.894953E−8	0.037134	46.219481	1.388088	7.204711E−7	2.000000	2.036003E−7	1.563853
WOA	0.760149	0.000000E+0	0.036631	59.893379	1.739188	0.000000E+0	1.771903	3.017843E−7	1.474781
GA	0.761496	5.076614E−7	0.032608	79.732486	1.903717	6.103647E−7	1.970579	4.688430E−7	1.532605
WSO	0.760818	9.846510E−7	0.037358	57.848790	1.988027	1.173585E−7	1.399368	5.345950E−7	1.895039

**Table 17:** Summary of root mean square error (RMSE) results obtained by competing algorithms for the TDM.

Algorithm	Best	Worst	Mean	STDEV
CPO_DE	7.474295E−4	7.975950E−4	7.639980E−4	6.741994E−6
CPO	7.397604E−4	2.715746E−3	1.150797E−3	4.486298E−4
DE	7.610084E−4	2.173161E−3	9.037716E−4	2.362304E−4
PSO	7.584090E−4	2.220808E−3	1.311558E−3	3.430002E−4
GWO	8.397812E−4	4.283961E−2	6.867118E−3	1.061972E−2
DPGSK	7.593799E−4	2.235170E−3	1.230806E−3	2.637327E−4
SAO	7.564424E−4	6.229928E−3	2.266435E−3	1.163250E−3
HOA	9.292243E−4	8.024784E−3	3.158337E−3	1.171341E−3
GOA	8.948558E−4	3.628282E−2	8.526781E−3	7.369645E−3
WOA	8.891981E−4	4.846594E−2	1.234308E−2	1.501588E−2
GA	1.823148E−3	4.254282E−3	3.213685E−3	4.615249E−4
WSO	7.441565E−4	2.101919E−3	1.014413E−3	2.167167E−4

**Fig. 17:**
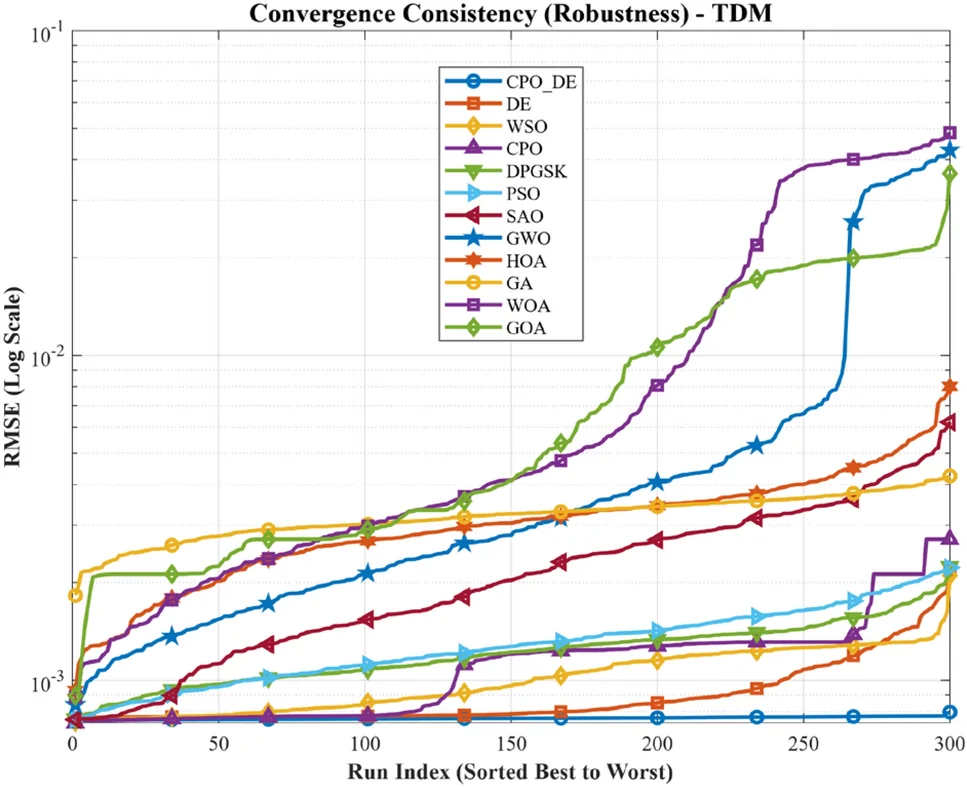
Stability and robustness of the evaluated algorithms for the TDM.

**Fig. 18:**
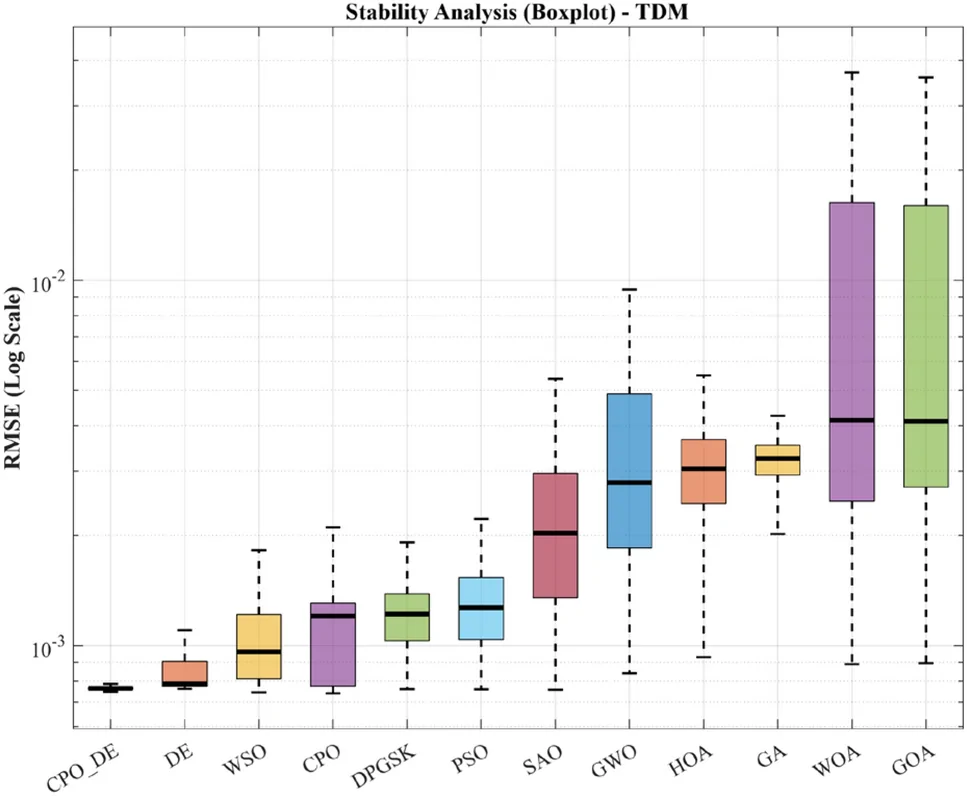
Stability analysis (Boxplot) for the TDM.

**Fig. 19:**
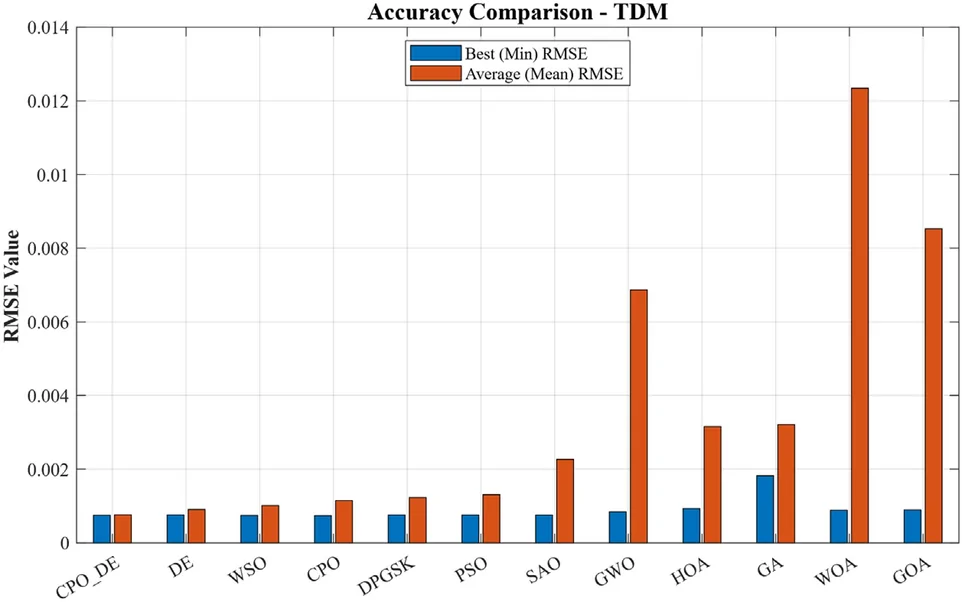
Accuracy comparison for the TDM.

**Fig. 20:**
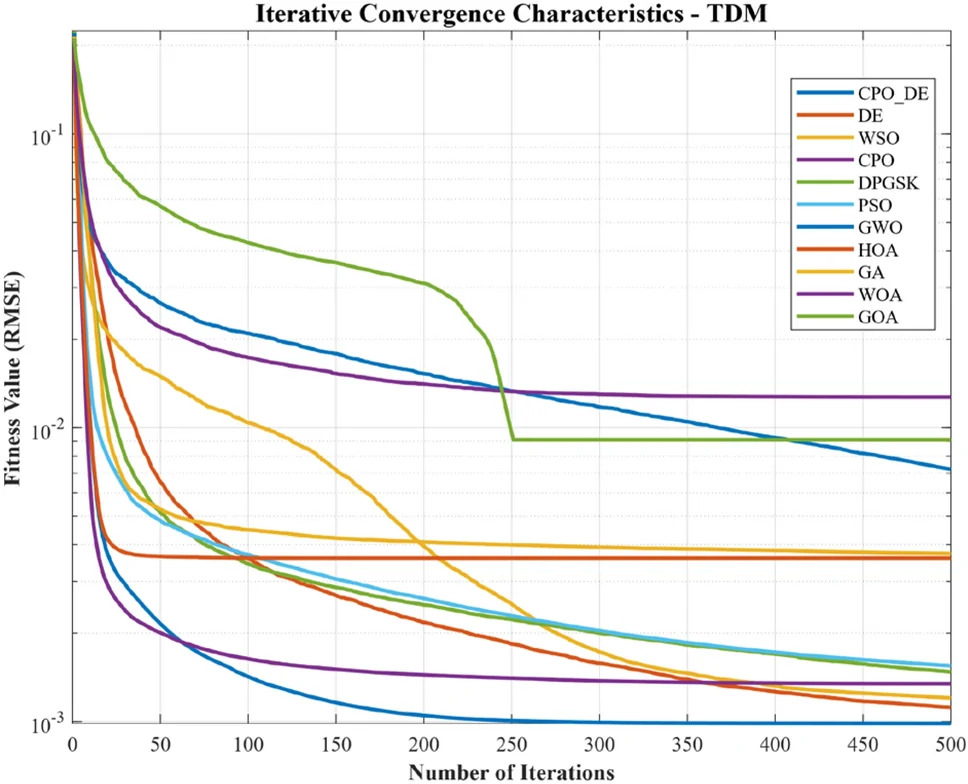
Iterative convergence characteristics of the evaluated algorithms for the TDM.

The main advantage of CPO-DE in this complex model is its high stability. Achieving a low standard deviation of $6.741\times {10}^{-6}$, CPO-DE's "Worst" RMSE $\left(7.975\times {10}^{-4}\right)$ is notably better than the "Mean" RMSE of almost all competing algorithms. This consistency is supported by the steady robustness curve Fig. 17, the compact variance in the boxplot Fig. 18, and the minimal best-to-mean accuracy gap Fig. 19. Furthermore, the iterative convergence curve Fig. 20 demonstrates the algorithm's dynamic efficiency, confirming a rapid and steady error descent across iterations without premature stagnation.

Non-parametric statistical tests confirm these results. Like the DDM case, CPO-DE outperformed all 11 competitors in the Wilcoxon rank-sum test Table 18. Finally, the Friedman mean rank test Table 19 ranks CPO-DE first with a score of 1.2867, proving its effectiveness and reliability in solving the highly complex TDM.

**Table 18:** Wilcoxon rank-sum test (compared to baseline: CPO-DE) for the TDM.

Algorithm	p_value	Decision
CPO_DE	1.00000E+00	-
DE	3.12384E−83	Loss (−)
WSO	8.16820E−75	Loss (−)
CPO	7.71441E−53	Loss (−)
DPGSK	2.45126E−98	Loss (−)
PSO	3.15824E−97	Loss (−)
SAO	3.06358E−91	Loss (−)
GWO	1.04970E−99	Loss (−)
HOA	1.04970E−99	Loss (−)
GA	1.04970E−99	Loss (−)
WOA	1.04970E−99	Loss (−)
GOA	1.04970E−99	Loss (−)

**Table 19:** Friedman mean rank test (lower is better) for the TDM.

Final position	Algorithm	Mean rank
Rank 1	CPO_DE	1.2867
Rank 2	DE	3.1067
Rank 3	WSO	3.6967
Rank 4	CPO	4.0100
Rank 5	DPGSK	5.0533
Rank 6	PSO	5.3333
Rank 7	SAO	7.4167
Rank 8	GWO	9.0433
Rank 9	HOA	9.2100
Rank 10	GA	9.5633
Rank 11	WOA	10.0800
Rank 12	GOA	10.2000

## Conclusions

This paper presented a novel hybrid optimization algorithm, CPO-DE, to accurately estimate the parameters of solar PV models. By combining the global exploration capabilities of the Crested Porcupine Optimizer (CPO) with the stable mutation mechanisms of Differential Evolution (DE), the proposed method avoids premature convergence and stagnation in local optima. Before its application to PV systems, CPO-DE was extensively evaluated on 23 standard mathematical benchmark functions and three conventional constrained engineering problems (tension/compression spring, welded beam, and pressure vessel designs). The results demonstrated an effective balance between exploration and exploitation, outperforming the original CPO across diverse search spaces.

The algorithm was subsequently applied to the Single, Double, and Triple Diode Models (SDM, DDM, and TDM) using experimental data from the RTC France solar cell. To enhance computational efficiency, a multi-stage evaluation strategy was introduced. The integration of fast algebraic equations during the iterative search loop, followed by the exact Newton–Raphson solver at the final stage, reduced computational time while maintaining physical precision. Consequently, the parameters estimated by CPO-DE produced simulated I-V and P–V characteristic curves that closely aligned with the experimental data under standard operating conditions.

Statistically, the proposed method demonstrated high stability, achieving highly consistent convergence to the optimal solutions across all independent runs. It recorded near-zero standard deviations, such as $8.104\times {10}^{-12}$ for the SDM, confirming its reliability compared to standard stochastic algorithms. Even in the complex 9-dimensional TDM, CPO-DE maintained its performance, consistently outperforming eleven other optimization algorithms and ranking first in the Friedman statistical tests.

In conclusion, the CPO-DE framework provides a computationally efficient, stable, and accurate tool for modern solar PV modeling. However, while highly effective for moderate-dimensional PV models, its convergence speed and exploration capabilities may degrade in complex, high-dimensional problems due to the curse of dimensionality.

To bridge the gap between theoretical optimization and practical applications, future work will focus on deploying CPO-DE on low-cost microcontrollers. By minimizing its memory footprint and computational complexity, the algorithm will be adapted for real-time Maximum Power Point Tracking (MPPT) to efficiently track the global peak under dynamic Partial Shading Conditions (PSC).

## Supplementary Information

## Data Availability

The data that support the findings of this study are available from the corresponding author upon reasonable request.

## Abbreviations

CPO: Crested porcupine optimizer
CPO-DE: Hybrid crested porcupine differential evolution
DDM: Double Diode Model
DE: Differential evolution
NFL: No free lunch
NR: Newton–Raphson
PV: Photovoltaic
RMSE: Root mean square error
SDM: Single Diode Model
TDM: Triple Diode Model

## Symbols

V: Measured output voltage of the PV cell/module (V)
I: Measured output current of the PV cell/module (A)
${I}_{calc}$: Simulated (calculated) current using the explicit solver (A)
${I}_{ph}$: Photo-generated current (A)
${I}_{sd1}$: Reverse saturation current of the first diode (A)
${I}_{sd2}$: Reverse saturation current of the second diode (A)
${I}_{sd3}$: Reverse saturation current of the third diode (A)
${n}_{1}$: Ideality factor of the first diode (–)
${n}_{2}$: Ideality factor of the second diode (–)
${n}_{3}$: Ideality factor of the third diode (–)
$${R}_{s}$$: Series resistance (*Ω *)
${R}_{sh}$: Shunt (parallel) resistance (*Ω *)
*q*: Electron charge $$(1.602\times {10}^{-19})$$ (C)
*k*: Boltzmann constant $$(1.38\times {10}^{-23})$$ (J/K)
*T*: Absolute temperature of the p–n junction (K)
