# Supplementary material for: A hybrid metaheuristic framework for robust photovoltaic model parameter estimation based on crested porcupine and differential evolution algorithms
Source: Sci Rep. 2026 Sep 2;16:27556. doi: 10.1038/s41598-026-67591-x (PMC13538469; doi:10.1038/s41598-026-67591-x)
Supplement: Supplementary file 1 [file 41598_2026_67591_MOESM3_ESM.docx]

A Hybrid Metaheuristic Framework for Robust Photovoltaic Model Parameter Estimation Based on Crested Porcupine and Differential Evolution Algorithms (Supplementary Material)

Ali Ahmed Hussein Mohamed ^1,^<https://orcid.org/0009-0005-4349-1908>, Ibrahim Mohamed Diaaeldin ^2,^<https://orcid.org/0000-0002-8720-4780>, Mahmoud A. Attia ^1,^ *<https://orcid.org/0000-0002-6603-7434> and Walid Helmy ^1^

^1^	Electrical Power and Machines Department, Faculty of Engineering, Ain Shams University, Cairo 11517, Egypt; [2300741@eng.asu.edu.eg](mailto:2300741@eng.asu.edu.eg) (A.A.H.M.), [mahmoud.abdullah@eng.asu.edu.eg](mailto:mahmoud.abdullah@eng.asu.edu.eg) (M.A.A.), [walid_helmy@eng.asu.edu.eg](mailto:walid_helmy@eng.asu.edu.eg) (W.H.)

^2^	Engineering Physics and Mathematics Department, Ain Shams University, Cairo 11517, Egypt; [ibrahimmohamed@eng.asu.edu.eg](mailto:ibrahimmohamed@eng.asu.edu.eg) (I.M.D.)

*****	Correspondence: [mahmoud.abdullah@eng.asu.edu.eg](mailto:mahmoud.abdullah@eng.asu.edu.eg) (M.A.A.)

Supplementary Note 1: Mathematical Formulations of Benchmark Functions

To thoroughly evaluate the mathematical robustness of the proposed CPO-DE algorithm prior to its engineering application, a standard set of 23 benchmark test functions was utilized ^1^^,^^2^. As outlined in Table A1, these functions are systematically categorized into three distinct groups to assess different search capabilities:

- Unimodal functions $(f_{1}-f_{7})$: Characterized by a single global optimum, these functions are specifically utilized to evaluate the pure local exploitation capacity and convergence speed of the algorithm.
- Multimodal functions $(f_{8}-f_{13})$: Containing numerous local optima, these functions are designed to assess the global exploration ability and the algorithm's effectiveness in avoiding premature convergence.
- Fixed-dimension multimodal functions $(f_{14}-f_{23})$: Featuring lower dimensions but highly complex mathematical landscapes, these functions rigorously test the overall stability and precision of the optimization process.

The complete mathematical formulations, problem dimensionality $(n)$, variable search space boundaries, and the theoretical global minimum objective values for all 23 functions are comprehensively summarized in Table 1.

**Table 1** Mathematical formulations of the 23 standard benchmark functions.

| **Objective function** | **Description** | $\boldsymbol{n}$ | **Variables’ limits** | **Minimum objective** |
| --- | --- | --- | --- | --- |
| Uni-modal test functions | | | | |
| $f_{1}$ | $\sum_{i=1}^{n} x_{i}^{2}$ | 30 | [-100, 100] | 0 |
| $f_{2}$ | $\sum_{i=1}^{n} \left\vert x_{i} \right\vert+\prod_{i=1}^{n} \left\vert x_{i} \right\vert$ | 30 | [-10, 10] | 0 |
| $f_{3}$ | $\sum_{i=1}^{n} \left( \sum_{j=1}^{i} x_{j} \right)^{2}$ | 30 | [-100, 100] | 0 |
| $f_{4}$ | $\max_{i} \left\{ \left\vert x_{i} \right\vert,1\leq i\leq n \right\}$ | 30 | [-100, 100] | 0 |
| $f_{5}$ | $\sum_{i=1}^{n} \left( 100\left( x_{i+1}-x_{i}^{2} \right)^{2}+\left( x_{i}-1 \right)^{2} \right)$ | 30 | [-30, 30] | 0 |
| $f_{6}$ | $\sum_{i=1}^{n} \left( x_{i}+0.5 \right)^{2}$ | 30 | [-100, 100] | 0 |
| $f_{7}$ | $\sum_{i=1}^{n} ix_{i}^{4}+random[0,1)$ | 30 | [-1.28, 1.28] | 0 |
| Multi-modal test functions | | | | |
| $f_{8}$ | $\sum_{i=1}^{n} {-x}_{i}\sin(\sqrt{\left\vert x_{i} \right\vert})$ | 30 | [−500, 500] | −418.9829 × D |
| $f_{9}$ | $\sum_{i=1}^{n} \left( x_{i}^{2}-10\cos\left( 2\pi x_{i} \right)+10 \right)$ | 30 | [−5.12, 5.12] | 0 |
| $f_{10}$ | $-20\exp\left( -0.2\sqrt{\frac{1}{n}\sum_{j=1}^{n} x_{j}} \right)-\exp\left( \frac{1}{n}\cos\left( 2\pi x_{j} \right) \right)+20+e$ | 30 | [−32, 32] | 0 |
| $f_{11}$ | $\frac{1}{4000}\sum_{i=1}^{n} x_{i}^{2}-\prod_{i=1}^{n} \cos\left( \frac{x_{i}}{\sqrt{i}} \right)+1$ | 30 | [−600, 600] | 0 |
| $f_{12}$ | $\frac{\pi}{n}\left( 10\sin\left( \pi y_{1} \right)+\sum_{i=1}^{n} \left( y_{i}-1 \right)^{2}\left( 1+10\sin^{2} \left( \pi y_{i+1} \right) \right)+\left( y_{n}-1 \right)^{2} \right)+\sum_{i=1}^{n} u\left( x_{i},10, 100, 4 \right)$  $y_{i}=1+\frac{x_{i}+1}{4}$  $u\left( x_{i},a, k, m \right)=\left\{ \begin{aligned} k\left( x_{i}-a \right)^{m}, x_{i}>a \\ 0, -a<x<a \\ k\left( -x_{i}-a \right)^{m}, x_{i}<-a \end{aligned} \right.$ | 30 | [−50, 50] | 0 |
| $f_{13}$ | $0.1\left( 10\sin^{2} \left( 3\pi x_{1} \right)+\sum_{i=1}^{n} \left( x_{i}-1 \right)^{2}\left( 1+\sin^{2} (3\pi x_{i}+1 \right))+\left( x_{n}-1 \right)^{2}\left( 1+\sin^{2} \left( 2\pi x_{n} \right) \right) \right)+\sum_{i=1}^{n} u\left( x_{i},5, 100, 4 \right)$ | 30 | [−50, 50] | 0 |
|  | | | | |
| $f_{14}$ | $\left( \frac{1}{500}+\sum_{i=1}^{25} \frac{1}{i+\sum_{j=1}^{2} \left( x_{i}-a_{ij} \right)^{6}} \right)^{-1}$ | 2 | [−65, 65] | 1 |
| $f_{15}$ | $\sum_{i=1}^{11} \left( a_{i}-\frac{x_{1}\left( b_{i}^{2}+b_{i}x_{2} \right)}{b_{i}^{2}+b_{i}x_{3}+x_{4}} \right)^{2}$ | 4 | [−5, 5] | 0.00030 |
| $f_{16}$ | $4x_{1}^{2}-2.1x_{1}^{4}+\frac{1}{3}x_{1}^{6}+x_{1}x_{2}-4x_{2}^{2}+4x_{2}^{4}$ | 2 | [−5, 5] | −1.0316 |
| $f_{17}$ | $\left( x_{2}-\frac{5.1}{4\pi^{2}}x_{1}^{2}+\frac{5}{\pi}x_{1}-6 \right)^{2}+10\left( 1-\frac{1}{8\pi} \right)\cos x_{1}+10$ | 2 | [−5, 5] | 0.398 |
| $f_{18}$ | $\left( 1+\left( x_{1}+x_{2}+1 \right)^{2}\left( 19-14x_{1}+3x_{1}^{2}-14x_{2}+6x_{1}x_{2}+3x_{2}^{2} \right) \right)\left( 30+\left( 2x_{1}-3x_{2} \right)^{2}\left( 18-32x_{1}+12x_{1}^{2}+48x_{2}-36x_{1}x_{2}+27x_{2}^{2} \right) \right)$ | 2 | [−2, 2] | 3 |
| $f_{19}$ | $-\sum_{i=1}^{4} c_{i}\exp\left( -\sum_{j=1}^{3} a_{ij}\left( x_{j}-p_{ij} \right)^{2} \right)$ | 3 | [1, 3] | −3.86 |
| $f_{20}$ | $-\sum_{i=1}^{4} c_{i}\exp\left( -\sum_{j=1}^{6} a_{ij}\left( x_{j}-p_{ij} \right)^{2} \right)$ | 6 | [0, 1] | −3.32 |
| $f_{21}$ | $-\sum_{i=1}^{5} \left( \left( X-a_{i} \right)\left( X-a_{i} \right)^{T}+c_{i} \right)^{-1}$ | 4 | [0, 10] | −10.1532 |
| $f_{22}$ | $-\sum_{i=1}^{7} \left( \left( X-a_{i} \right)\left( X-a_{i} \right)^{T}+c_{i} \right)^{-1}$ | 4 | [0, 10] | −10.4028 |
| $f_{23}$ | $-\sum_{i=1}^{10} \left( \left( X-a_{i} \right)\left( X-a_{i} \right)^{T}+c_{i} \right)^{-1}$ | 4 | [0, 10] | −10.5363 |

Supplementary Note 2: Mathematical Formulations of Classical Engineering Applications

Following the comprehensive evaluation of the proposed CPO-DE framework on benchmark functions and PV parameter estimation, it is essential to further verify its robustness and versatility in handling complex real-world constraints. Therefore, in this section, the proposed CPO-DE algorithm is tested on three well-known classical constrained engineering design problems ^3^: (a) Tension/compression spring design problem, (b) Welded beam design problem, and (c) Pressure vessel design problem. The mathematical formulations and constraints of these problems are given as follows:

1. **Tension/compression spring design problem**

| $x=\left[ x_{1} x_{2} x_{3} \right]=\left[ d D N \right]$  Minimize:  $f\left( x \right)=x_{1}^{2}x_{2}\left( x_{3}+2 \right)$  Subject to:  $g_{1}\left( x \right)=1-\frac{x_{2}^{3}x_{3}}{71785x_{1}^{4}}\leq0$  $g_{2}\left( x \right)=\frac{4x_{2}^{2}-x_{1}x_{2}}{12566x_{1}^{3}\left( x_{2}-x_{1} \right)}+\frac{1}{5108x_{1}^{2}}-1\leq0$  $g_{3}\left( x \right)=1-\frac{140.45x_{1}}{x_{3}x_{2}^{2}}\leq0$  $g_{4}\left( x \right)=\frac{x_{1}+x_{2}}{1.5}-1\leq0$  $0.05\leq x_{1}\leq2$  $0.25\leq x_{2}\leq1.3$  $2\leq x_{3}\leq15$ | (1) |
| --- | --- |

2. **Welded beam design problem**

| $x=\left[ x_{1} x_{2} x_{3} x_{4} \right]=\left[ h l t b \right]$  Minimize:  $f\left( x \right)=1.10471x_{1}^{2}x_{2}+0.04811x_{3}x_{4}\left( 14+x_{2} \right)$  Subject to:  $g_{1}\left( x \right)=\tau-\tau^{max}\leq0$  $g_{2}\left( x \right)=\sigma-\sigma^{max}\leq0$  $g_{3}\left( x \right)=\delta-\delta^{max}\leq0$  $g_{4}\left( x \right)=x_{1}-x_{4}\leq0$  $g_{5}\left( x \right)=P-P^{C}\leq0$  $g_{6}\left( x \right)=0.125-x_{1}\leq0$  $g_{7}\left( x \right)=0.10471x_{1}^{2}+0.04811x_{3}x_{4}\left( 14+x_{2} \right)-5\leq0$  $0.1\leq x_{1}\leq2$  $0.1\leq x_{2}\leq10$  $0.1\leq x_{3}\leq10$  $0.1\leq x_{4}\leq2$  $P=6000 lb, L=14 in, E=30\times{10}^{6} psi, G=12\times{10}^{6} psi$  $\tau^{max}=13600 psi, \sigma^{max}=30000 psi, \delta^{max}=0.25 in$  $M=P\left( L+\frac{x_{2}}{2} \right),R=\sqrt{\frac{x_{2}^{2}}{4}+\left( \frac{x_{1}+x_{3}}{2} \right)^{2}},\tau^{'}=\frac{P}{\sqrt{2x_{1}x_{2}}}, \tau^{''}=\frac{MR}{J}$  $J=2\sqrt{2}x_{1}x_{2}\left( \frac{x_{2}^{2}}{12}+\left( \frac{x_{1}+x_{3}}{2} \right)^{2} \right), \tau=\sqrt{\tau^{'2}+\frac{\tau^{'}\tau^{''}x_{2}}{R}+\tau^{''2}}$  $P^{C}=4.013\frac{E}{L^{2}}\sqrt{\frac{x_{3}^{2}x_{4}^{6}}{36}}\left( 1-\frac{x_{3}}{2L}\sqrt{\frac{E}{4G}} \right)$  $\sigma=\frac{6PL}{x_{4}x_{3}^{2}}, \delta=\frac{4PL^{3}}{Ex_{4}x_{3}^{2}}.$ | (2) |
| --- | --- |

3. **Pressure vessel design problem**

| $x=\left[ x_{1} x_{2} x_{3} x_{4} \right]=\left[ T_{s} T_{h} R L \right]$  Minimize:  $f\left( x \right)=0.6224x_{1}x_{3}x_{4}+1.7781x_{2}x_{3}^{2}+3.1661x_{1}^{2}x_{4}+19.84x_{1}^{2}x_{3}$  Subject to:  $g_{1}\left( x \right)=-x_{1}+0.0193x_{3}\leq0$  $g_{2}\left( x \right)=-x_{2}+0.00954x_{3}\leq0$  $g_{3}\left( x \right)=-\pi x_{3}^{2}x_{4}-\frac{4}{3}\pi x_{3}^{2}+1296000\leq0$  $g_{4}\left( x \right)=x_{4}-240\leq0$  $0\leq x_{1}\leq99$  $0\leq x_{2}\leq99$  $10\leq x_{3}\leq200$  $10\leq x_{4}\leq200$ | (3) |
| --- | --- |

Supplementary References

1	Diaaeldin, I. M., Hasanien, H. M., Qais, M. H., Alghuwainem, S. & Omar, O. A. M. Multi scenario chaotic transient search optimization algorithm for global optimization technique. *Sci. Rep.* **15**, 4284 (2025). <https://doi.org/10.1038/s41598-025-86757-7>

2	Yao, X., Liu, Y. & Lin, G. Evolutionary programming made faster. *IEEE Trans. Evol. Comput.* **3**, 82–102 (1999). <https://doi.org/10.1109/4235.771163>

3	Qais, M. H., Hasanien, H. M. & Alghuwainem, S. Transient search optimization: a new meta-heuristic optimization algorithm. *Applied Intelligence* **50**, 3926-3941 (2020). <https://doi.org/10.1007/s10489-020-01727-y>
